# Hyperspectral Imaging and Machine Learning for Automated Pest Identification in Cereal Crops

**DOI:** 10.3390/biology14121715

**Published:** 2025-12-01

**Authors:** Rimma M. Ualiyeva, Mariya M. Kaverina, Anastasiya V. Osipova, Alina A. Faurat, Sayan B. Zhangazin, Nurgul N. Iksat

**Affiliations:** 1 Department of Biology and Ecology, Toraighyrov University, Pavlodar 140008, Kazakhstan; ualiyeva.r@gmail.com (R.M.U.); aanastasiyaaa@internet.ru (A.V.O.); 2 Department of Geography and Tourism, Toraighyrov University, Pavlodar 140008, Kazakhstan; alina03.09@mail.ru; 3 Department of Biotechnology and Microbiology, L.N. Gumilyov Eurasian National University, Astana 010000, Kazakhstan; sayanzhangazin@gmail.com (S.B.Z.); nurguliksat@gmail.com (N.N.I.)

**Keywords:** hyperspectral imaging, insect pests, spectral characteristics, remote sensing, wheat agrocenosis, agricultural monitoring

## Abstract

The spectral characteristics of harmful insect pests in wheat fields were characterised using hyperspectral imaging for the first time. The analysis of spectral profiles revealed that reflectance is determined by the structure of the insect’s chitin and the colouration of its body surface. Insects with lighter or more vivid colours (white, yellow, or green) showed higher reflectance values compared to those with predominantly dark pigmentation. Reflectance was also influenced by the presence of wings, surface roughness, and the age of the insect. Each species exhibited distinct spectral patterns that allowed for differentiation not only from other insect species but also from the plant background. A classification model using PLS-DA was developed and demonstrated high accuracy in identifying 12 pest species, confirming the strong potential of hyperspectral imaging for species-level classification. The results validate the PLS-DA method for differentiating insects based on spectral characteristics and underscore the reliability of this approach for automated monitoring systems to detect phytophagous pests in crop fields. This technology could reduce insecticide use by 30–40% through targeted application. The research has both scientific and economic significance, laying the groundwork for integrating machine learning and computer vision into agricultural monitoring. It supports the advancement of precision farming and contributes to improved global food security.

## Simple Summary

Timely detection of insect pests is critical for protecting crops and ensuring food security. This study explores the potential of hyperspectral imaging, an advanced remote sensing technology, for the accurate identification of various insect pest species within wheat agrocenoses. We found that different insect species possess unique spectral signatures, driven by differences in colouration, surface structure, and morphometric characteristics. Leveraging these distinctions, we were able to classify 12 pest species with high accuracy using machine learning algorithms. Notably, species with distinct colouration or pronounced structural features—such as light-coloured bugs or insects with transparent wings—were identified most reliably. In contrast, smaller or dark-coloured species showed slightly lower classification accuracy. A key advantage of our method is its ability to differentiate even closely related species, enabling the development of automated drone-based monitoring systems. Our findings confirm that hyperspectral imaging can serve as a powerful tool for eco-friendly and precise pest control, enabling farmers to apply protective measures in a targeted manner. This approach enhances the efficiency of pest management strategies but also helps reduce negative environmental impacts.

## 1. Introduction

Hyperspectral imaging is a method of proximal sensing [1,2]. It records from several dozen to several hundred narrow spectral channels (typically 200–300 within the 400–2500 nm range), forming a continuous spectrum for each image pixel [3,4,5]. This method generates a continuous spectrum for each pixel in an image, enabling the identification of materials and objects through the integrated analysis of spatial and spectral information within a unified framework [6,7,8]. Consequently, hyperspectral imaging reveals fine details of objects and facilitates their classification [9].

Portable hyperspectral systems show significant promise for field applications, particularly when mounted on Unmanned Aerial Vehicles (UAVs) for agricultural monitoring [10,11,12]. Beyond applications in mining, oil and ore prospecting, hyperspectral imaging is increasingly used to analyse plant diseases, food products, environmental pollution, and material quality control. It enables assessment of the quality of freshly harvested products during storage and processing, which are prone to rapid deterioration due to oxidation, moisture loss, and nutrient depletion. The technology can detect changes in colour, texture, and defects [13], as well as monitor the presence of pathogenic bacteria such as *Escherichia coli* and *Listeria monocytogenes* [14,15], along with viruses and fungi [16].

For the analysis of biological objects, the most informative spectral ranges are the visible (400–700 nm) and near-infrared (700–1100 nm) regions. The former is primarily used for assessing pigmentation, while the latter provides information on the structural characteristics of tissues [17,18]. In addition, the short-wave infrared range (1100–2500 nm) is mainly used for identifying chemical composition [19,20].

In entomology, hyperspectral imaging facilitates the identification of closely related insect species [21], morphological studies, and field surveys to detect crops or plants infested with harmful insects [22,23,24,25]. Such studies have demonstrated species identification accuracies of 92–95% using machine learning methods applied to hyperspectral data [26]. Previous studies have utilised imaging systems to analyse cuticle and phenotypic changes in *Sitophilus oryzae* [27] and classify *Anastrepha fraterculus*, *Anastrepha obliqua*, *Anastrepha sororcula Zucchi*, *Drosophila melanogaster*, *Drosophila simulans*, *Heliothis virescens*, and *Helicoverpa zea*. This technology has also been used to detect *Tetramorium caespitum*, *Tetramorium impurum*, *Trichogramma* spp., and infestations of *Cryptolestes ferrugineus* inside wheat grains [28].

A rapidly advancing application is the integration of hyperspectral systems with UAVs to monitor crops and map the distribution of key pest species [29,30,31,32,33]. This approach can identify infestation hotspots with over 85% accuracy [34]. Specific studies have successfully employed hyperspectral imaging to identify *Tetranychus urticae* [35] and *Hemiptera Pseudococcidae* [36] in cotton, describe the integration of alpha taxonomy, mitochondrial DNA and hyperspectral reflectance profiles of *Cicadellidae* [37]. Further applications include identifying *Halyomorpha halys* [38], studying *Orthoptera* taxonomy [39], and researching agricultural pests in Central China [40]. The technology makes it possible to identify similar species of crickets [41], study the structure of insects [42], and their optical properties of the structural colouration of the elytra [43]. For example, near-light reflectivity is expressed in white, red, orange, and yellow insects and has also been found in green insects (e.g., the leaf beetles *Ancylecha fenestrata*, *Stipnochlora couloniana*, and the walking leaf beetle *Phyllium celebicum*). The metallic-green wings of the butterfly *Trides priamus* and the elytra of *Chrysaspis aurovittata* are non-reflective in near-light [44].

A primary application of hyperspectral imaging is object classification [45], including the detection and differentiation of biological agents such as pathogens, pests, and fungal infections [46,47,48]. This is particularly important in agriculture, where accurate and timely recognition of pest infestations allows for crop protection measures [49] and can improve the accuracy and efficiency of food quality control.

Therefore, the aim of our study was to investigate the phenotypic characteristics and identify harmful entomofauna in wheat agrocenoses using hyperspectral imaging and computer vision. We specifically aimed to: (1) acquire and analyse the reflectance spectra of key phytophagous pests, (2) decipher their unique spectral profiles for identification, and (3) develop and validate a classification model to assess the reliability of this method for pest monitoring. We hypothesise that the combination of hyperspectral imaging in combination with computer vision technologies enables highly accurate identification of harmful entomofauna by identifying the characteristic spectral signatures of phytophages, facilitating rapid monitoring and timely crop protection measures.

## 2. Materials and Methods

### 2.1. Research Objects and Test Sites

The following insect species were selected as study objects: *Anisoplia austriaca*, *Anisoplia agricola*, *Phorbia fumigata*, *Trigonotylus ruficornis*, *Phyllotreta vittula*, *Haplothrips tritici*, *Chorosoma schillingii*, *Loxostege sticticalis*, *Tettigonia viridissima*, *Chaetocnema aridula*, *Calliptamus italicus*, *Laodelphax striatella*.

The classification of these species and the rationale for their selection are presented in Table 1.

**Figure 1:**
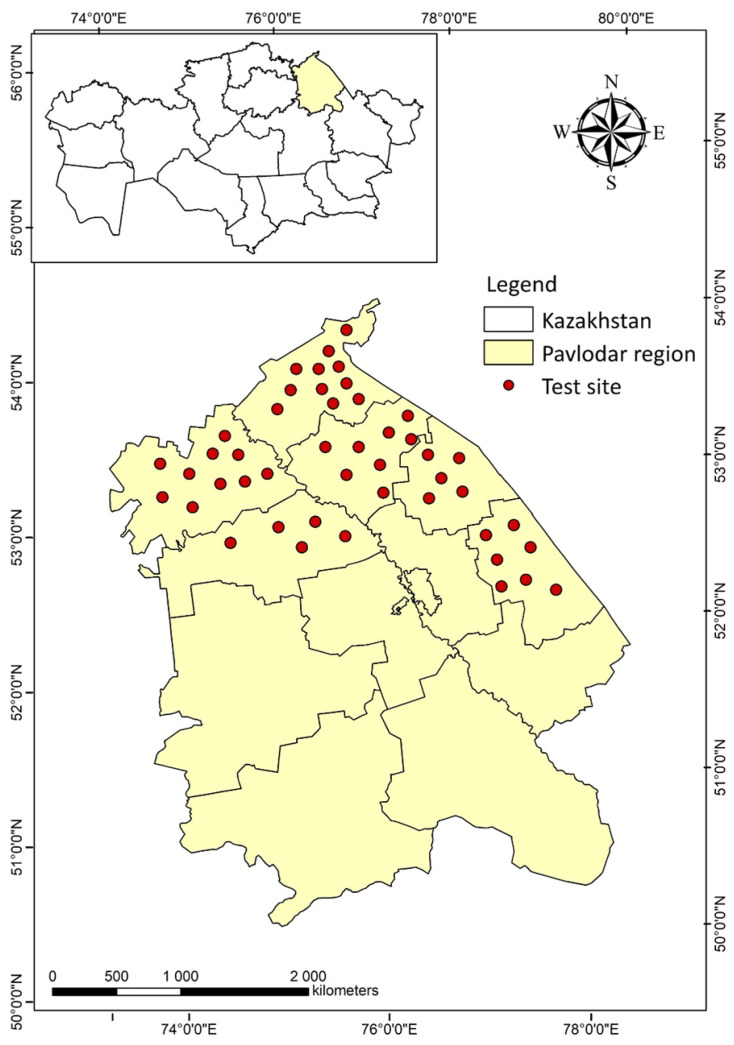
Main grain-growing regions of the Pavlodar region (northeastern Kazakhstan; test sites).

Insects were sampled from spring wheat crops during the 2025 growing season in the main grain-growing regions of northeastern Kazakhstan (Figure 1).

Field studies were conducted in the northeastern part of Kazakhstan, within the Pavlodar region (52.2–52.5° N, 76.8–77.1° E), in areas under spring wheat cultivation. The plots were located in typical steppe agro-landscapes, characterised by flat terrain with slight topographical differentiation.

The region has a sharply continental climate, with cold winters and hot, predominantly dry summers. During the 2025 growing season (May–August), weather conditions were moderately warm with uneven precipitation.

May was characterised by cool and windy conditions, with an average daily air temperature of +13–14 °C and precipitation not exceeding 15–20 mm. In June, temperatures steadily increased to +19–20 °C, with limited precipitation (~25 mm), leading to partial soil drought at the beginning of tillering and stem elongation. July and August received significantly more precipitation than the long-term average: approximately 65 mm in July and 70 mm in August, mainly in the form of short but intense showers. The average temperature in July was +22 °C and in August +20 °C, with relative humidity ranging from 55 to 65%. Thus, rainfall in the second half of the summer contributed to the stabilisation of soil moisture, positively affecting plant condition and the abundance of phytophagous insects during the heading-ripening phase.

Predominant winds were from the southwest and northeast, with speeds of 3–5 m/s. The duration of sunshine from May to August exceeded 1200 h, promoting intensive biomass accumulation and insect development.

Soils were predominantly dark-chestnut and chestnut medium loams. Soils contain 2.5–3.0% organic matter, have a calcareous profile, slightly alkaline reaction (pH 7.3–7.6), and good water permeability. Temporary drying was observed in the upper horizons in June, but soil moisture improved in July–August due to rainfall. Low-lying areas showed signs of temporary waterlogging. The reserves of productive moisture in the 0–50 cm soil layer were 60–80 mm at the beginning of the growing season, decreased to 30–40 mm in June, and recovered to 70–90 mm by the end of July.

### 2.2. Collection and Taxonomic Identification of Entomological Material

Insects were collected from spring wheat crops during the 2025 growing season. Sampling was carried out using the route method to ensure uniform coverage of the study areas.

Insects were sampled along transects during the active vegetation phases of spring wheat (BBCH 55–75). Each route was approximately 2.5 km long, with ten fixed sampling points spaced every 250 m. Collection and visual inspections were conducted during daytime hours (10:00–15:00) under clear weather conditions. Air temperature during the surveys ranged from +22 to +26 °C, relative humidity was 45–55%, and wind speed did not exceed 3 m/s.

Insects were collected using an entomological net (rim diameter 35–40 cm, handle length 1–1.2 m) from the vegetative parts of the plants, as well as manually from ears and leaves. A quantitative count of pests was conducted using a standard entomological technique [50]: 100 sweeps with a standard net (diameter 30 cm, bag depth 60 cm, handle length 1 m) were made in the upper grass layer.

Collected insects were euthanized with ethyl acetate vapour in a portable killing jar. Each specimen was labelled with collection data and transported to the laboratory for further morphological examination and hyperspectral analysis.

During the 2025 field surveys in six major grain-growing regions of northeastern Kazakhstan, approximately 350 specimens of harmful insects were collected, representing 12 species of the wheat agrocenosis entomofauna (*A. austriaca*—25, *A. agricola*—28, *Ph. fumigata*—30, *Tr. ruficornis*—38, *Ph. vittula*—35, *H. tritici*—55, *Ch. schillingii*—25, *L. sticticalis*—24, *T. viridissima*—20, *Ch. aridula*—22, *C. italicus*—25, *L. striatella*—23). The most abundant species in the sample was *H. tritici* (wheat thrips), with a population of up to 55 individuals. *Ph. vittula* and *Tr. ruficornis* were also frequently encountered, with 35–38 specimens of each species. Collected specimens were preserved and transported to the laboratory for hyperspectral imaging within two hours of collection to prevent changes in body colouration due to drying.

Taxonomic identification was conducted using a stereoscopic microscope (×10–×40 magnification) based on diagnostic features (antennae structure, head shape, wing venation, colour, setae, and integument characteristics). We used specialised identification keys [51] for agricultural insects to confirm species.

### 2.3. Hyperspectral Imaging

All laboratory studies were conducted at the Biological Research Laboratory of Toraighyrov University (Pavlodar, Kazakhstan).

#### 2.3.1. Sample Preparation for Hyperspectral Imaging

Insect specimens were prepared for hyperspectral imaging according to a standardised protocol. Prior to data acquisition, imaging panels with a smooth, matte surface were prepared to minimise light reflection and facilitate accurate calibration. Insect samples were positioned 3–5 cm apart to prevent overlap and ensure precise segmentation during subsequent image processing. The distance between the specimens and the hyperspectral camera was maintained at 30–40 cm, depending on the insect’s size, the lens used, and the desired spatial resolution.

Insects were fixed in a natural position—primarily dorsal side up (e.g., *A. austriaca*, *A. agricola*, *L. sticticalis*, *H. tritici*, *Ph. vittula*, *Ch. aridula*, *Tr. ruficornis*, *Ch. schillingi*, *L. striatella*) or lateral side up (*Ph. fumigata*, *C. italicus*, *T. viridissima*) to ensure full access to the dorsal, lateral, and ventral surfaces for detailed imaging of external morphology. Fixation was carried out with minimal mechanical interference to preserve the natural colouration and integrity of the insect integument.

Immediately before imaging, the hyperspectral camera was calibrated using a Spectralon white reference standard and a dark reference to ensure radiometric accuracy and eliminate system noise. The Spectralon panel, made from a fluoropolymer with high diffuse reflectance in the visible and near-infrared ranges, served as a standard for white balance calibration [52].

#### 2.3.2. Hyperspectral Imaging and Image Processing

##### Technical Parameters and Imaging Mode

Hyperspectral imaging was performed using a FigSpec FS-13 VNIR scanning camera (400–1000 nm) (Hangzhou, China) employing a linear push-broom scanning method. The system uses a transmission diffraction grating as its spectral dispersive element and is capable of recording up to 1200 spectral bands with a spectral resolution of 2.5 nm. Illumination was provided by two LED light sources with a colour temperature of 5500 K and a colour rendering index (CRI) > 95, creating uniform irradiance of approximately 800 lux on the sample surface. The light incidence angle was 45°, preventing glare and ensuring stable reflectance measurements. Before each series of scans, radiometric calibration was performed using a Spectralon reference plate (99%) (Labsphere, Inc., North Sutton, NH, USA). The distance from the lens to the sample was 25 cm, corresponding to a spatial resolution of 0.3 mm/pixel. The spatial resolution of the sensor is 1920 pixels per line, with a pixel size of 5.86 µm—an essential parameter for capturing fine morphological details of small insects. The camera is equipped with a CMOS detector, which ensures high sensitivity and low noise levels, both critical for reliable spectral analysis. Image acquisition was carried out at a frame rate of 128 frames per second in full spectral mode, allowing continuous data collection across the entire VNIR range (400–1000 nm). This enabled a comprehensive analysis of reflectance and absorption characteristics of various insect morphological structures [53].

##### Image Processing and Preparation for Classification

The acquired hyperspectral images were processed to extract reflectance spectra of the insect specimens using Breeze software (licenced version 2024.2.0) with support for the IDL programming language, which allows for automated and customisable processing of large hyperspectral datasets. The image data were handled in the form of hyperspectral cubes—three-dimensional arrays combining spatial and spectral information.

Image preprocessing included radiometric and spectral calibration using reference images from white and black panels to calculate correction coefficients. Spatial masking was then performed to remove background and non-relevant pixels, thereby eliminating the influence of external reflections, noise, and glare. Masks were generated using Breeze’s ROI tools, as well as through binarisation based on spectral homogeneity. Clustering was performed using the k-means algorithm with 3–5 clusters, and morphological filtering was applied using opening and closing operations with a structural element of 3-pixel radius.

Spectral data extracted from the masked regions were subjected to further processing, including smoothing, Standard Normal Variate (SNV) normalisation, and centring. These procedures reduced the influence of noise and systematic errors, enhancing the information content of the spectral features for subsequent classification tasks.

#### 2.3.3. Interactive Spectral Analysis Using PCA and Pixel Explore

To analyse the spectral characteristics of insect samples, we used the Pixel Explore tool. This tool allows us to interactively select areas directly on the hyperspectral image by using a PCA variance scatter plot. After selecting a region, the sample is added to the training dataset and labelled accordingly. The visualisation interface includes three main parts: Raw Spectrum—shows light reflectance across different wavelengths (up to 1000 nm) for each insect sample; Variance Scatter plot—displays how individual pixels are distributed along two principal components (t[1] and t[2]) that capture the main spectral differences; Max Variance image—highlights the image area with the most spectral variation. In the scatter plot, each dot represents a single pixel from the image. Dots that are close together indicate similar spectral properties. The colour of the dots reflects pixel density—red means more pixels with similar characteristics. The first principal component (t[1]) captures the most noticeable differences between spectra and is useful for grouping insects with similar spectral traits. The second component (t[2]) picks up on variations in surface texture and fine morphological features. The Max Variance image, automatically generated by the Breeze software (version 2024.2.0), is essentially a snapshot of the hyperspectral data at the wavelength where the differences between samples are most distinct. This helps to identify quickly the most informative regions for further analysis.

#### 2.3.4. Machine Learning for Identifying and Differentiating Pest Species

The system was trained to detect, differentiate, and identify pest types by extracting and generating new feature vectors based on the combination and optimisation of spectral characteristics, followed by input into machine learning classification algorithms. In the “Model” menu, the statistical Sample model was selected, where the Principal Component Analysis (PCA) method is applied, and background pixels are removed from the image in such a way as to preserve the relevant Region of Interest (ROI).

Three main analytical and machine learning approaches were used:

- PCA (Principal Component Analysis)—to reduce the number of spectral variables while keeping the most important differences;
- PLS-DA (Partial Least Squares Discriminant Analysis)—to classify samples based on spectral data;
- SVM (Support Vector Machine) with a radial basis function (RBF) kernel and default parameter settings for C and γ, as implemented in Breeze, was used to perform both binary and multi-class classification tasks [33].

**Figure 2:**
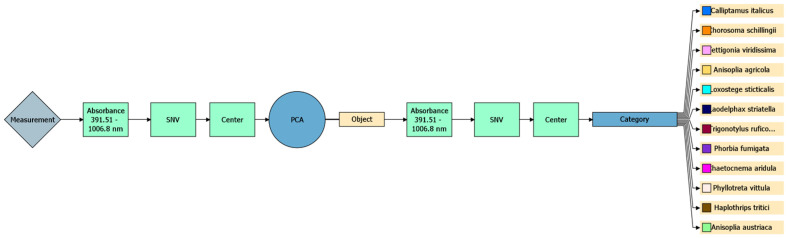
Spectral data and class labels used as input parameters for building the classification model.

The overall process is illustrated in Figure 2:

The hyperspectral data was collected in the 391.51–1006.8 nm range, covering both visible and near-infrared (VNIR) regions [10]. Although this goes slightly beyond the official range of the camera’s sensor, the sensor still captures usable data at the extremes. Using the full range—even where signal quality is lower—helped improve classification accuracy.

To train the model, we manually labelled subsets of pixels according to insect species, body parts, and tissue types. Model performance was evaluated using cross-validation, with standard metrics such as accuracy, recall, and F1-score.

### 2.4. Statistical Data Processing

Statistical analysis, including analysis of variance (ANOVA), was performed to validate the obtained results. These methods allow us to evaluate the reliability and accuracy of the classification outcomes. ANOVA and descriptive statistics were applied to process the spectral data.

The minimum and maximum reflectance values were calculated using equation:

R_min_ = min(R_1_, R_2_, …, R_n_), (1)

R_max_ = max(R_1_, R_2_, …, R_n_), (2)

where Rₘᵢₙ is the minimum reflectance value in the sample, Rₘₐₓ is the maximum reflectance value, R_1_ to Rₙ are individual reflectance measurements, and n is the total number of measurements.

The mean reflectance value (μ), indicating the overall reflectance of the object within the studied spectral range, was calculated using equation:

(3) $$\mu =\frac{1}{n}\sum_{i=1}^{n}R_{i},$$

where Rᵢ is the reflectance value for the i-th measurement, and n is the total number of measurements [54].

The standard deviation (σ) represents the dispersion of reflectance values from the mean, providing insight into the variability within the sample. It was calculated using equation:

(4) $$\sigma =\sqrt{\frac{1}{n}\sum_{i=1}^{n}{{(R}_{i}-\mu )}^{2}},$$

where μ is the arithmetic mean of the reflectance coefficient, Rᵢ is the reflectance coefficient value for the i-th measurement, and n is the total number of measurements [54].

The coefficient of variation (CV) quantifies relative variability, expressing the extent to which the reflectance values deviate from the mean as a percentage. It was calculated using equation:

(5) $$CV=\frac{\sigma }{\mu }\times 100,$$

where σ is the standard deviation, and μ is the mean reflectance value [54].

The reflectance delta (ΔR) was calculated using equation to represent the difference between the maximum and minimum reflectance values in a given spectral range or under different conditions:

(6) $$∆R=R_{max}-R_{min}$$

where Rₘᵢₙ is the minimum reflectance coefficient value, and Rₘₐₓ is the maximum reflectance coefficient value [54].

The spectral bandwidth (SB) reflects the width of the spectral range where significant changes in reflectance occur. It helps identify regions with distinct optical properties, such as maximum absorption or reflection. It was calculated using equation:

(7) $$SB=\lambda_{max}-\lambda_{min},$$

where λₘᵢₙ and λₘₐₓ are the minimum and maximum wavelengths, respectively [55].

The spectral asymmetry (SA) indicates the skewness in the distribution of reflectance values and was calculated using equation:

(8) $$SA=\frac{1}{n}\sum_{i=1}^{n}{\left(\frac{R_{i}-\mu }{\sigma }\right)}^{3},$$

where σ is the standard deviation, μ is the arithmetic mean of the reflectance coefficient, Rᵢ is the reflectance coefficient value for the i-th measurement, and n is the total number of measurements [56].

The calculated parameters—including minimum and maximum reflectance, mean reflectance, standard deviation, and coefficient of variation—enable a quantitative assessment of the spectral characteristics of the studied objects and facilitate the identification of differences among them.

To evaluate the performance of predictive models developed using hyperspectral data, the coefficient of determination (R^2^), the predictive ability of the model (Q^2^), and the root mean square error of calibration (RMSEC) were computed.

The coefficient of determination (R^2^), which indicates the proportion of variance in the dependent variable explained by the model, was calculated using equation:

(9) $$R^{2}=1-\frac{\sum_{i=1}^{n}{\left(y_{i}-{\overset{ˇ}{y}}_{i}\right)}^{2}}{\sum_{i=1}^{n}{\left(y_{i}-\bar{y}\right)}^{2}},$$

where $y_{i}$ are the true values, ${\overset{ˇ}{y}}_{i}$ are the predicted values, $\bar{y}$ is the mean of the true values, and n is the number of observations [57].

The predictive ability of the model (Q^2^), calculated on a test set, was determined using equation:

(10) $$Q^{2}=1-\frac{\sum_{i=1}^{n}{\left(y_{i}^{test}-{\overset{ˇ}{y}}_{i}^{test}\right)}^{2}}{\sum_{i=1}^{n}{\left(y_{i}^{test}-{\bar{y}}^{train}\right)}^{2}},$$

where $y_{i}^{test}$ and ${\overset{ˇ}{y}}_{i}^{test}$ are the actual and predicted values for the test set, and ${\bar{y}}^{train}$ is the mean of the training set values [57].

The root mean square error of calibration (RMSEC), indicating the deviation of the model’s predictions from actual values in the training set, was calculated using equation:

(11) $$RMSEC=\sqrt{\frac{1}{n}\sum_{i=1}^{n}{\left(y_{i}-{\overset{ˇ}{y}}_{i}\right)}^{2}},$$

where $y_{i}$ are the true values (in the training dataset), ${\overset{ˇ}{y}}_{i}$ are the values predicted by the model, and n is the number of observations [57].

These statistical metrics form the basis for further development of classification models and can be used for effective identification and monitoring of pests in agricultural fields, thereby enhancing crop protection strategies.

## 3. Results

**Figure 3:**
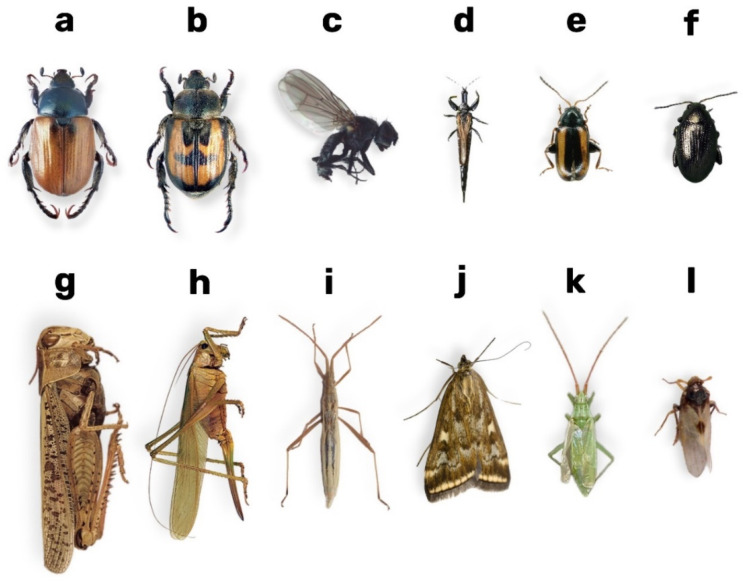
Main study objects ((**a**)—*Anisoplia austriaca*, (**b**)—*Anisoplia agricola*, (**c**)—*Phorbia fumigata*, (**d**)—*Haplothrips tritici*, (**e**)—*Phyllotreta vittula*, (**f**)—*Chaetocnema aridula*, (**g**)—*Calliptamus italicus*, (**h**)—*Tettigonia viridissima*, (**i**)—*Chorosoma schillingii*, (**j**)—*Loxostege sticticalis*, (**k**)—*Trigonotylus ruficornis*, (**l**)—*Laodelphax striatella*).

Figure 3 presents photographs of the insects analysed in this study, which are crucial for understanding their morphological structure.

The images presented below illustrate the spectral data of insects obtained using hyperspectral imaging. On the right are the hyperspectral images of the specimens, while on the left are the Raw Spectrum graph and the Variance Scatter plot. The Raw Spectrum graph contains several curves, each representing an individual insect sample or a specific part of it. In the hyperspectral image, the insect samples are numbered in accordance with the curves. For example, the first red line in the Raw Spectrum graph corresponds to sample number one in the hyperspectral image. For some samples, an additional Raw Spectrum graph is provided separately to demonstrate the spectra of particular body parts.

To describe the obtained data, the following terms will be used: dark and light (in relation to pigmentation), high and low (in relation to reflectance), and dull and bright (in relation to the colouration of insect body parts). This system of definitions allows for a generalised description of the observed characteristics influencing spectral parameters and facilitates the interpretation of results in the Discussion section.

### 3.1. Spectral Characteristics of Insect Morphological Structures and Integuments Based on Principal Component Analysis (PCA)

**Figure 4:**
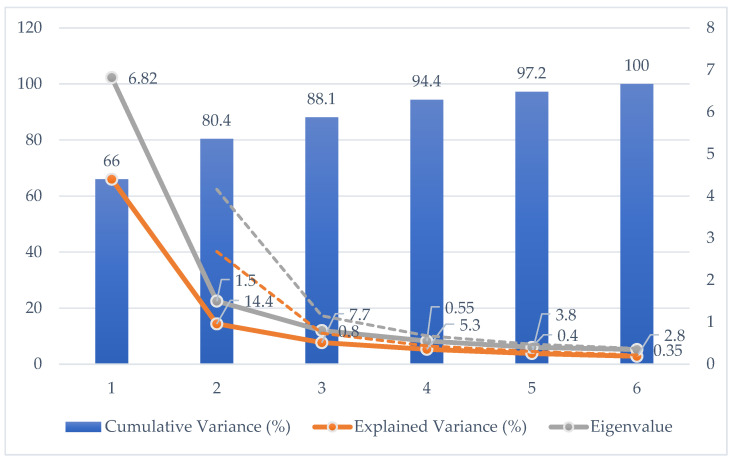
Scree plot of principal components.

In order to justify the reduction to two principal components, we performed a PCA on the spectral data of pests. Table 2 presents the eigenvalues, explained variance, and cumulative variance for the first six principal components. Figure 4 shows the corresponding scree plot.

The scree plot and cumulative variance indicate that the first two principal components explain 80.4% of the total variance in the dataset, which represents the majority of the spectral variability. Based on this, we considered two principal components sufficient for subsequent analyses, as adding additional components would provide only marginal increases in explained variance while increasing model complexity.

#### 3.1.1. *Anisoplia austriaca* and *Anisoplia agricola*

This study analyses two beetle species from the genus *Anisoplia*: *Anisoplia austriaca* and *Anisoplia agricola*. *A. austriaca* has a convex, oval-shaped body measuring 12–18 mm in length, with dark (black, grey-black), metallic colour. The brown elytra are rectangular and feature a black scutellum at the base. *A. agricola* has a similar body shape, but the elytra display distinctive cross-shaped black markings.

The raw spectral plot displays reflectance curves in the 500–800 nm wavelength range for all samples. A primary reflectance peak occurs in the 600–750 nm region, and a secondary peak appears between 750 and 800 nm, indicating minimal reflectance in the near-infrared (NIR) region. This reduced reflectance is attributed to the light-absorbing properties of body areas rich in melanin.

Spectra two, six, and eight correspond to *A. austriaca* and exhibit the highest intensity (over 48%) due to the reflective nature of the brown elytra. Specimen six shows the brightest surface, with reflectance values exceeding 60%. In contrast, spectra one, three, and five represent *A. agricola*, which displays moderate reflectance (~37–42%) due to pigmented, cross-shaped markings that enhance light absorption and reduce reflectance. Spectral differences may also reflect variations in the smoothness of the chitinous surface.

**Figure 5:**
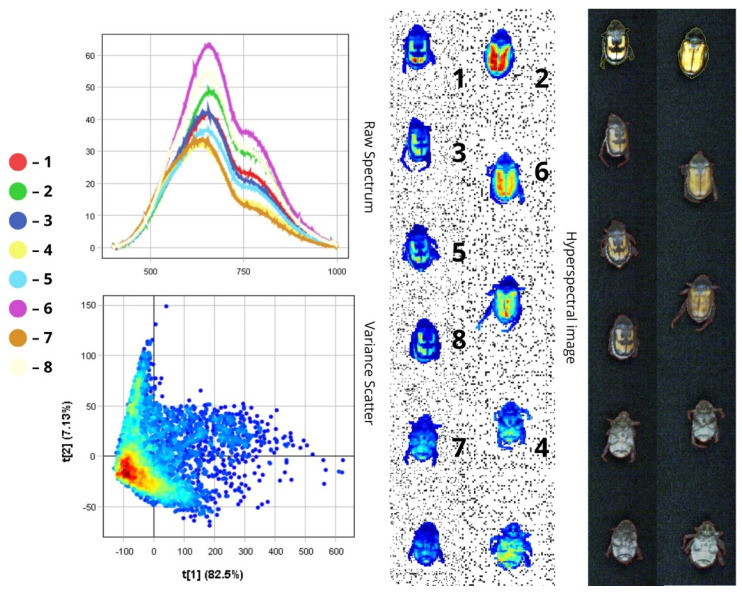
Spectral characteristics of Anisoplia beetles extracted from the hyperspectral image.

The fourth and seventh spectra represent specimens with the dark abdomen facing upward. These body regions absorb more light than they reflect, resulting in lower intensity (~33–34%). Additionally, the rougher texture of the abdomen, often covered with fine hairs, further reduces reflectance—particularly in the VNIR region, where it can drop below 15% (Figure 5).

In the PCA scatter plot, the first principal component (t[1]) accounts for the majority of the variance (82.5%), indicating that most spectral variability lies along a single dimension. This results in a narrow distribution of points along the t[1] axis and a dense cluster in the central region. The second principal component accounts for only 7.13% of the variance. The high cluster density reflects the overall uniformity in the beetles’ surface structure and size.

In *Anisoplia* beetles, reflectance and absorption in the head, scutellum, and pronotum are primarily observed within the 500–750 nm range (visible spectrum). The spectra for these regions exhibit relatively uniform reflectance (~20%) due to stable pigmentation. However, slight deviations may arise from increased melanisation of the chitinous exoskeleton, potentially caused by pigment oxidation, tissue dehydration, or increased surface roughness. The PCA scatter plot confirms these variations are minimal.

**Figure 6:**
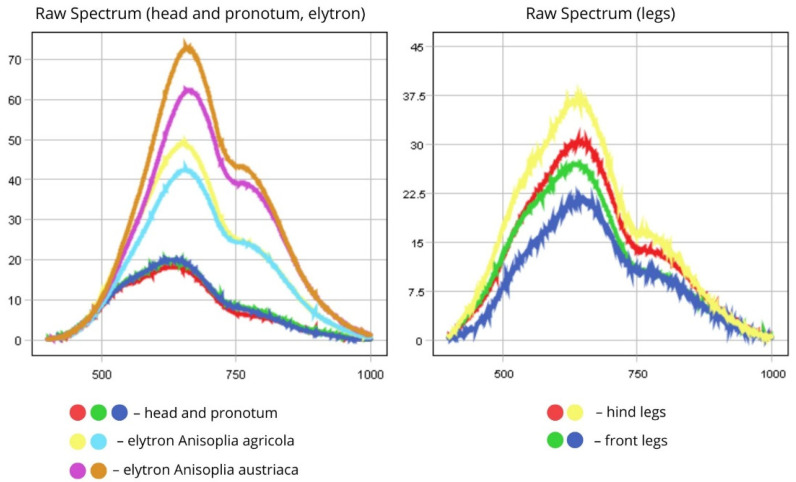
Spectral characteristics of individual morphological structures of *Anisoplia* beetles extracted from the hyperspectral image.

The elytral spectra show peak reflectance within the 600–800 nm range, encompassing both the visible (600–750 nm) and near-infrared (750–800 nm) regions. This indicates that the elytra reflect light effectively in the NIR range. *A. austriaca* displays higher reflectance (RC = 40–43%) than *A. agricola* (RC = ~25%), likely due to the absence of pigmented markings on its dorsal side, which are saturated with melanin. Intraspecific variations in spectral intensity may result from differences in elytral condition (e.g., thinning) and the specific region chosen for spectral measurement (Figure 6).

The limbs show their highest reflectance (reaching up to 37.5%) in the 500–750 nm range. However, the forelimbs exhibit lower reflectance values (RC = 21–27%) compared to the hindlimbs (RC = 30–37.5%). This discrepancy may be attributed to differences in morphology, texture, pigmentation, and orientation relative to the light source. In many insects, including species of *Anisoplia*, the hindlimbs are larger and may have a smoother or denser cuticle that reflects more light, resulting in higher spectral intensities (over 30%).

In contrast, the forelimbs are typically smaller and may possess a rougher surface, which scatters light and reduces reflectance. Additionally, reflectance values can vary due to differences in light incidence and reflection angles during hyperspectral imaging. Larger hindlimbs are often better orientated to reflect light efficiently, while forelimbs—being closer to the head and body—may be partially shaded, leading to lower reflectance values (below 28%).

#### 3.1.2. *Phorbia fumigata*

**Figure 7:**
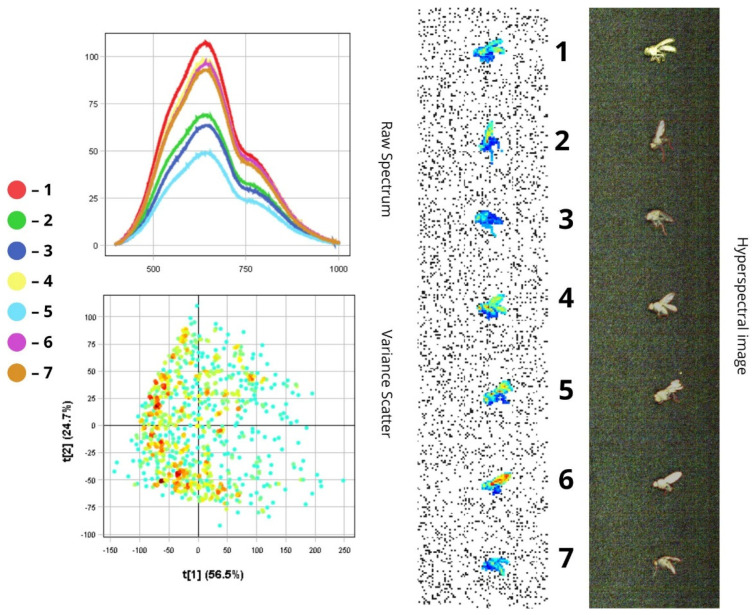
Spectral characteristics of *Phorbia fumigata* extracted from the hyperspectral image.

**Figure 8:**
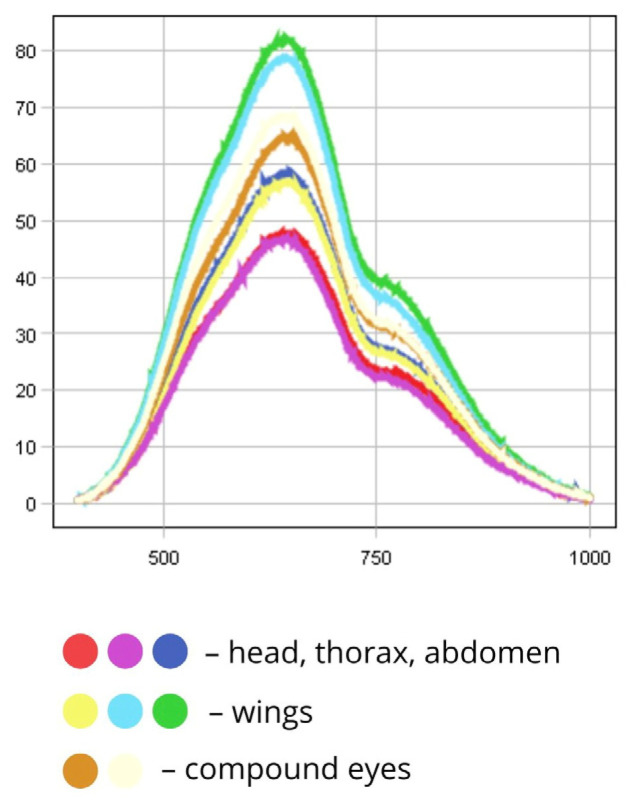
Spectral characteristics of body parts of *Phorbia fumigata*.

*Phorbia fumigata* is distinguished by its unique external features: a black body measuring between 5 and 8 mm in length and transparent wings with a grey or brownish tint. All spectral curves confirm a general reflectance pattern characteristic of this species, with intensity peaks in the 500–780 nm range, indicating NIR reflectance due to the wings’ reflective properties. The full spectral profile can be visually divided into two main zones:

(1) In the visible spectrum (400–700 nm), reflectance is relatively low (10–50%) (10–50%), which corresponds to the dark (below 50%), light-absorbing colouration of the insect’s body;
(2) In the near-infrared range, reflectance increases sharply, reaching peak values (Figure 7 and Figure 8).

Spectra one, four, six, and seven show higher reflectance (>85%) compared to others, likely due to specimen orientation that enhances wing surface exposure. The remaining spectra show reduced NIR intensity, which may result from both light absorption by dark pigments and increased surface roughness (Figure 7).

The wings exhibit spectral peaks within the 500–780 nm range, with high reflectance values in the NIR region. Their reflectance (RC ≈ 85%) exceeds that of other body regions. Variations in intensity may arise from differences in wing membrane thickness and insect positioning. The compound eyes demonstrate moderate reflectance (RC ≈ 65%), while the head, thorax, and abdomen display the lowest reflectance values due to pigment absorption (RC ≈ 48–60% in the visible range; RC ≈ 23–28% in the NIR) (Figure 8).

In the PCA scatterplot, pixel clustering shows that most of the fly’s surface exhibits similar spectral characteristics, suggesting surface homogeneity. The spread of pixels along the first principal component (PC1, 56.5%) reflects differences in overall luminance or total reflectance. Pixel displacement to the right (positive t[1] values) corresponds to highly reflective wing areas (over 85%), while negative t[1] values correspond to dark body regions (below 50%). The second principal component (PC2, 24.7%) captures more subtle spectral variations, likely resulting from differences in viewing angle or wing membrane thickness, which influence the shape of the spectral curve (Figure 7).

#### 3.1.3. *Calliptamus italicus*

*Calliptamus italicus* is typically 20–25 mm long and predominantly brown or yellowish-brown.

The spectral plot shows highest reflectance in specimens three and four, with peaks reaching 28–34.5% in the 550–750 nm range. Specimens one, two, and five exhibit the lowest reflectance, not exceeding 23–24%. The abdomens of specimens three, four, and five are visibly yellowish-red, while the others are positioned at different angles, making them appear darker. Peak reflectance is observed between 550 and 750 nm, with a notable decline beyond 800 nm in the NIR region, where reflectance drops to 10–15%.

**Figure 9:**
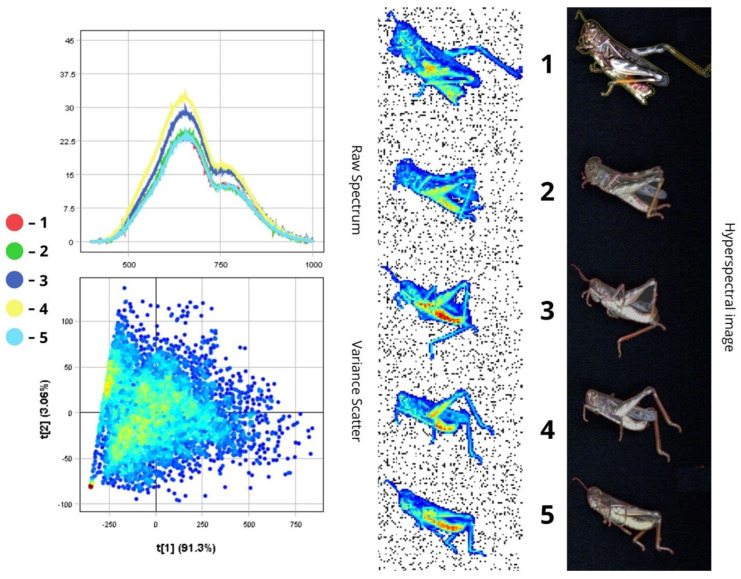
Spectral characteristics of *Calliptamus italicus* extracted from the hyperspectral image.

**Figure 10:**
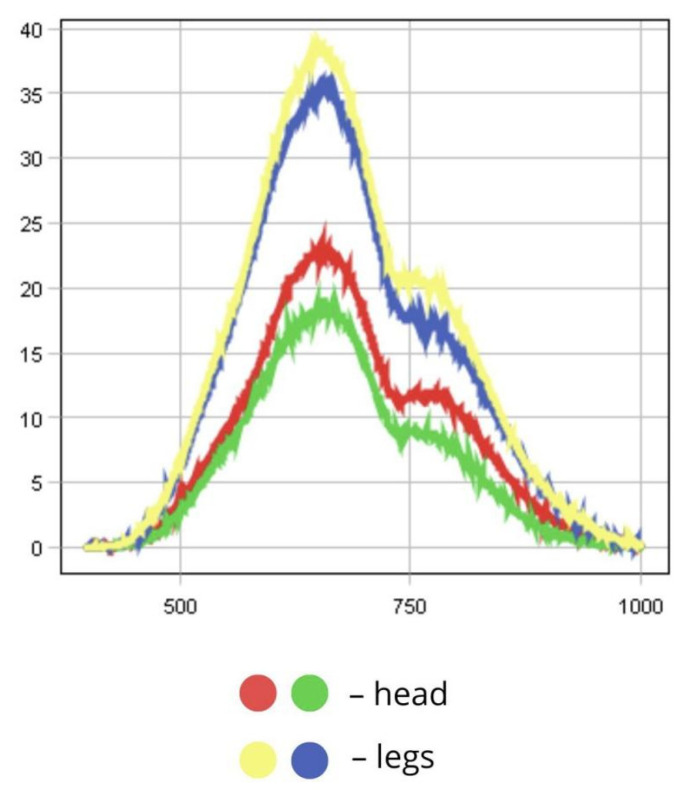
Spectral characteristics of body parts of *Calliptamus italicus*.

The raw spectrum plot illustrates reflectance data across all body parts, from head to limbs. The head appears darker both visually and spectrally due to high melanin content in the cuticle (over 30%). As melanin absorbs more light, reflectance is reduced in the corresponding body areas. This is shown in the graph: spectral curves covering the darker regions of the insect show significantly lower reflectance in the visible spectrum (19–23%) and a similar trend in the NIR range (9–12%). These darker features may serve camouflage purposes and provide UV protection. Melanin plays a photoprotective role by absorbing UV light, thereby preventing damage to vital cellular components such as lipids, proteins, and DNA. In addition, the lower reflectance of darker body regions (below 25%) may be due to the denser chitinous structure of the head and dorsal surfaces, potentially contributing to more effective thermoregulation. This can be advantageous in environments with fluctuating light conditions, helping reduce visibility to predators (Figure 9 and Figure 10).

In contrast, lighter-coloured extremities, which contain less pigment, show higher reflectance. Spectral curves for the limbs and lighter areas of the abdomen display a sharp increase in reflectance starting at ~500 nm, with peaks reaching 35–40% RC in the green-red region. This lighter pigmentation helps reflect sunlight, minimising the risk of overheating during active movement (Figure 10).

The dispersion plot reveals that most pixel variance is concentrated along the first principal component (PC1), which explains approximately 91.3% of the variability. This suggests that albedo (overall reflectance or brightness—22.5–34.5%) is the primary factor differentiating pixels. High positive values along the t[1] axis correspond to light-coloured body parts (abdomen and limbs), which have high reflectance (up to 40%), while dark morphological structures (RC below 23%) such as the head and dorsal surfaces correspond to negative t[1] values. The second principal component (PC2), which explains 9.06% of the variance, likely captures finer spectral variations, including differences in yellow and brown pigmentation across various body parts.

#### 3.1.4. *Loxostege sticticalis*

**Figure 11:**
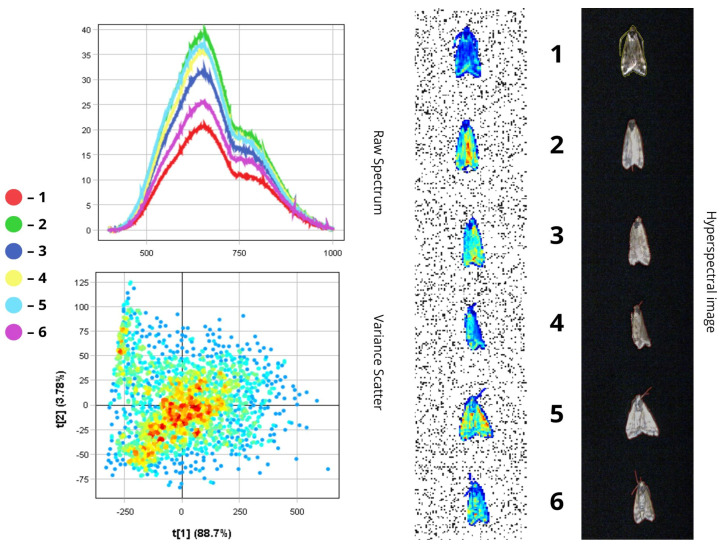
Spectral characteristics of *Loxostege sticticalis* extracted from the hyperspectral image.

*Loxostege sticticalis* has light brown forewings with a dark brown patterns and greyish-brown hindwings. The species has a head with compound eyes and filiform antennae, and a body length of ~17–19 mm (Figure 11).

The peak spectral intensity lies within the 500–780 nm range. The first specimen exhibits the lowest reflectance due to its darker brown colouration (RC = 20–21%), while the second specimen, with a lighter body, shows the highest reflectance values (R = 38–40%) (Figure 11).

According to the raw spectrum, the head reflects significantly less light than the wings, likely due to its darker pigmentation (RC = 13.5–18.5%). The wings demonstrate higher reflectance than other body parts due to their anatomical structure, which enhances reflection of incident light. Reflectance values for the wings range from 32% to 38.5%. This is facilitated by the presence of wing venation and the fine scales typical of *Lepidoptera*.

These wing scales may be pigmented, determining colour, or structural, producing optical effects such as iridescence or gloss via light interference, which enhances reflectance. Some scales possess microscopic structures that interact with light through reflection and refraction, significantly increasing wing reflectance even in the absence of bright pigmentation. The scale-covered wing surface is relatively smooth at the microscopic level, which further contributes to enhanced reflection.

Despite the presence of brown pigments, the light-coloured wings of *L. sticticalis* exhibit high reflectance (up to 38.5%). The thin, semi-transparent wing membrane also plays a role, resulting in greater reflectance compared to other body parts. High reflectance may serve as an adaptive trait—assisting in thermoregulation by reflecting solar radiation and aiding in camouflage by allowing the insect to blend into sunlit environments such as grass.

**Figure 12:**
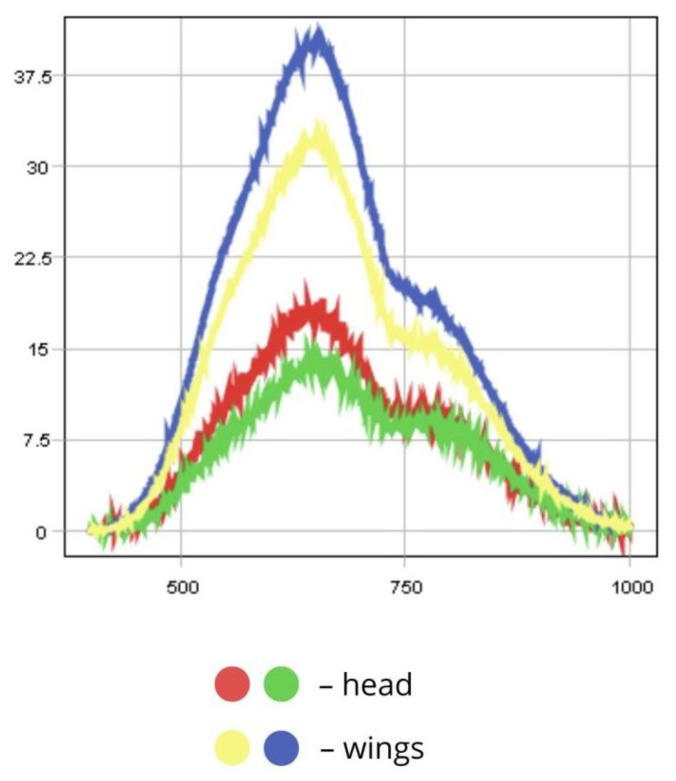
Spectral characteristics of body parts of *Loxostege sticticalis*.

The PCA scatterplot clearly shows separation of pixels along the first principal component (t[1]), which accounts for up to 88.7% of the total variance. The cluster of pixels with high positive t[1] values corresponds to wing areas with higher reflectance (32–38.5%), while the head, with lower reflectance (13.5–18.5%), is associated with negative t[1] values (Figure 12). This pronounced separation highlights reflectance intensity as a primary spectral feature for morphological differentiation.

#### 3.1.5. *Haplothrips tritici*

**Figure 13:**
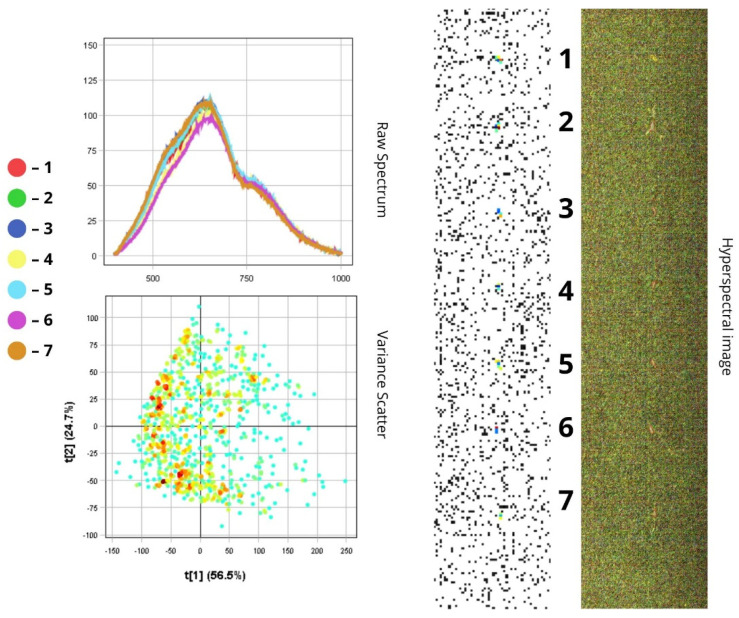
Spectral characteristics of *Haplothrips tritici* extracted from the hyperspectral image.

*Haplothrips tritici* is a small insect, measuring approximately 1–1.5 mm in length, with an elongated dark brown or black body marked by lighter stripes. The highest reflectance intensity (over 80%) is observed within the 500–780 nm range. Analysis of the raw spectral plot reveals that absolute reflectance values vary from 35 to 40% in darker pigmented areas to 80–90% in regions corresponding to wing edges and light body stripes. This creates a broad dynamic range of ~55–65 percentage points (Figure 13).

The PCA scatterplot shows PC1 accounts for ~56.5% of variance and describes this dispersion quantitatively, which is caused by a combination of the most important factors. This variability arises from multiple factors, including differences in chitin density and pigment concentration. Light-coloured body stripes increase reflectance, whereas higher pigmentation and denser cuticle result in lower spectral intensity (below 40%). Reflectance is also elevated in specimens oriented with their wings facing upward.

Thus, the dispersion indices shown in Figure 13 indicate that insect orientation and pigmentation level are the dominant factors shaping the structure of the principal components. These effects are further reinforced by physical and optical properties—such as cuticular texture and wing-edge structure—which create gradients in brightness and contribute to the observed geometric variability among samples. Additionally, the shape and tilt of the pixel cluster suggest that spectral differences are continuous, rather than discrete, as indicated by the smooth transitions between different zones in the scatterplot.

#### 3.1.6. *Phyllotreta vittula*

**Figure 14:**
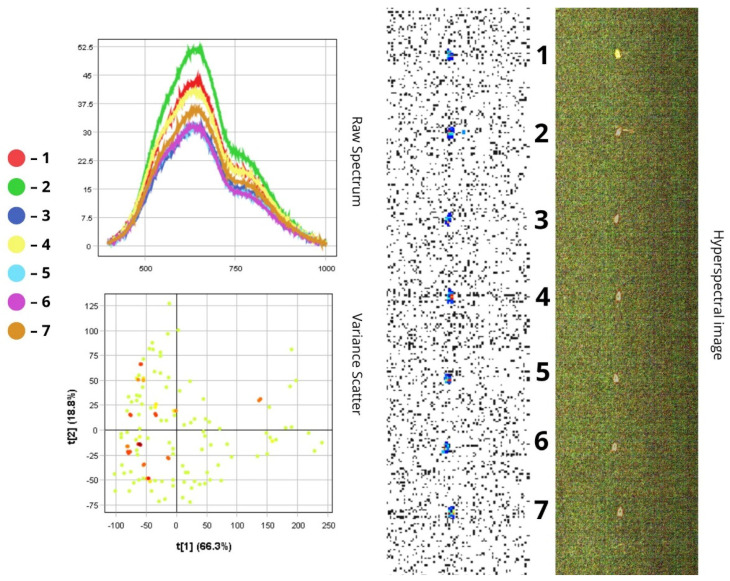
Spectral characteristics of *Phyllotreta vittula* extracted from the hyperspectral image.

*Phyllotreta vittula* is a small beetle (1–2 mm) with a black body and metallic green sheen on the head and pronotum, as well as a distinct yellow longitudinal stripe on each elytron. Hyperspectral analysis confirms this colour pattern: the yellow stripes typically produce a noticeable reflectance peak, particularly within the yellow-orange portion of the spectrum (~580–620 nm), while the metallic sheen results in a broader, flatter spectral curve across the visible range, in contrast to the matte black regions (Figure 14).

Spectral data for *P. vittula* show peak intensity within the 500–750 nm range. Reflectance values range from 15 to 20% in the darkest areas (e.g., black body parts) to 45–50% in brighter regions (e.g., yellow stripes), reaching up to 52.5% in the lightest areas. This demonstrates high spectral contrast despite the insect’s small size. A sharp drop in reflectance beyond 750 nm indicates reduced NIR reflectance of the chitinous exoskeleton. The highest reflectance spectra (over 50%) are likely associated with smoother, glossier surfaces and the light yellow elytral stripes. Conversely, lower reflectance values (~15%)—such as those observed in the third, fifth, and sixth spectra—may be due to the insect’s orientation (e.g., side view or dorsal exposure of the abdomen).

The PCA scatterplot reveals distinct clustering of pixel data. The first principal component (t[1]) accounts for 66.3% of the variance and clearly distinguishes between light and dark body regions: positive t[1] values correspond to bright elytral stripes (over 45%), while negative values represent the black body. The second component (t[2], 18.8%) likely reflects variation in the blue-green portion of the spectrum, characteristic of the metallic sheen, and helps separate pixels from the head and pronotum from those of the matte black abdomen (Figure 14).

#### 3.1.7. *Chaetocnema aridula*

*Chaetocnema aridula* is a small, oval-shaped insect measuring 2–3 mm in length. Its body has a shiny, greenish-bronze sheen, while the elytra, pronotum, and legs are uniformly black. The spectral profile confirms this uniformly dark colouration: all five spectral curves on the Raw Spectrum plot fall within a narrow range of values, demonstrating relatively low overall luminance. Reflectance in the visible range is between 10% and 19%, which is associated with high light absorption.

According to the Raw Spectrum plot, the low reflectance is due to the insect’s dark pigmentation, which absorbs a significant portion of incoming light. The reflectance peak lies within the 500–750 nm range, and the decline in intensity beyond 750 nm indicates reduced chitin reflectivity in the near-infrared region—a typical feature of dark-coloured surfaces. The highest reflectance (about 19%) was recorded for the first specimen, which is likely due to a smoother elytral surface. The reflectance peak shifts toward the blue-green region: reflectance begins to increase at 550 nm and gradually decreases by 750 nm. This spectral behaviour corresponds to the metallic sheen of the cuticle, reflecting the optical properties of an otherwise matte, dark surface. In contrast, the fourth specimen demonstrated the lowest reflectance, which is also due to its ventral-upward orientation that reduces the amount of reflected light.

**Figure 15:**
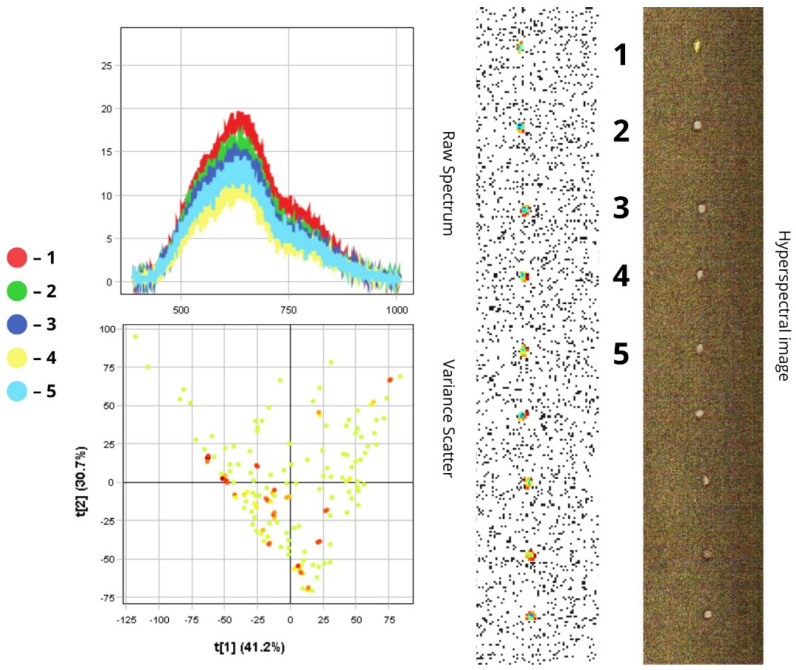
Spectral characteristics of *Chaetocnema aridula* extracted from the hyperspectral image.

The PCA scatterplot shows that the two principal components contribute relatively equally to total variance: PC1 (t[1]) accounts for 41.2%, and PC2 (t[2]) for 30.7%. This suggests that, unlike in species where brightness (RC about 17–19%) is the dominant factor, the spectral characteristics of this insect are shaped by two factors of comparable importance. Based on this, it can be assumed that t[1] encodes overall brightness variation, while t[2] reflects variability in metallic sheen across different body parts, depending on surface curvature (Figure 15). Scatterplots for *H. tritici*, *Ph. vittula*, and *Ch. aridula* exhibit relatively low pixel density, which is explained by the morphological features and spectral properties of these insects. Due to their small size, hyperspectral imaging captures a limited number of pixels, reducing the overall volume of spectral data. Additionally, the presence of pigments that absorb light in certain spectral ranges further reduces spectral variability, leading to lower scatter density in the data cloud.

#### 3.1.8. *Tettigonia viridissima*

**Figure 16:**
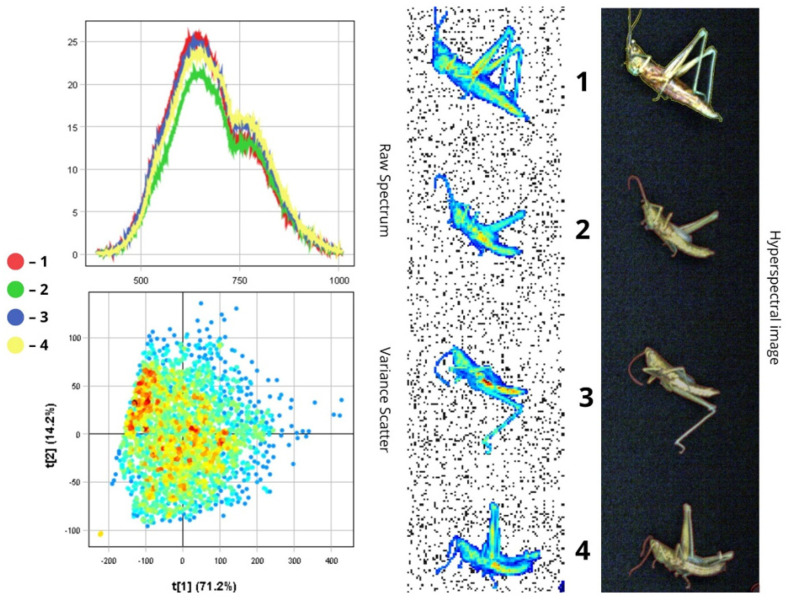
Spectral characteristics of *Tettigonia viridissima* extracted from the hyperspectral image.

Adult *Tettigonia viridissima* specimens typically measure 20–25 mm in length and exhibit a range of colours from green and yellow to pinkish-yellow (Figure 16). The head has a laterally compressed apex and long antennae. In males, the forewings feature a stridulatory organ, which includes a speculum—a transparent, resonant membrane—and a stridulatory area. This morphological polymorphism is reflected in the spectral data: although overall reflectance values do not differ greatly between samples, they range from 10% to 25% in the blue-green region (corresponding to darker green body areas) and up to 25% in the near-infrared region, particularly in lighter regions such as wing membranes.

The spectral peak falls within the 500–750 nm range, while a decline in intensity beyond 750 nm indicates reduced chitin reflectance

In the NIR range. According to the Raw Spectrum plot, the highest reflectance is observed in the first sample, which includes a wing region (RC = 25%). The wing membranes reflect more light than the chitinous exoskeleton. The lowest reflectance is recorded in the second sample (RC = 21%), where the wings are not visible. Wing size and position can significantly affect spectral intensity due to increased light scattering. This variation is seen in the difference between spectral curves: spectra from wing regions may display narrow, pronounced peaks around 550 nm (green region) with a sharp decline after 700 nm, whereas spectra from the body often exhibit flatter profiles, with peak reflectance shifting toward the yellow-orange zone (~600 nm).

The PCA scatterplot shows high point density, which is explained by the homogeneity of the cuticular surface and the large body size of *T. viridissima*. The first principal component (t[1]), accounting for 71.2% of the variance, stratifies the data by brightness (RC over 21%), separating pixels from the wings (positive t[1] values) and body (negative t[1] values). The second component (t[2]) likely reflects more subtle colour variations, such as gradients of yellow and green pigmentation. Due to the insect’s size, more spectral data are collected from different regions, increasing pixel density. However, spectral characteristics remain relatively consistent because of the structural similarity across large surface areas (Figure 16).

Thus, the variance ratio of the two principal components suggests that spectral diversity in this species is determined more by structural and optical properties (e.g., wings vs. exoskeleton) than by differences in pigmentation alone.

#### 3.1.9. *Trigonotylus ruficornis*

**Figure 17:**
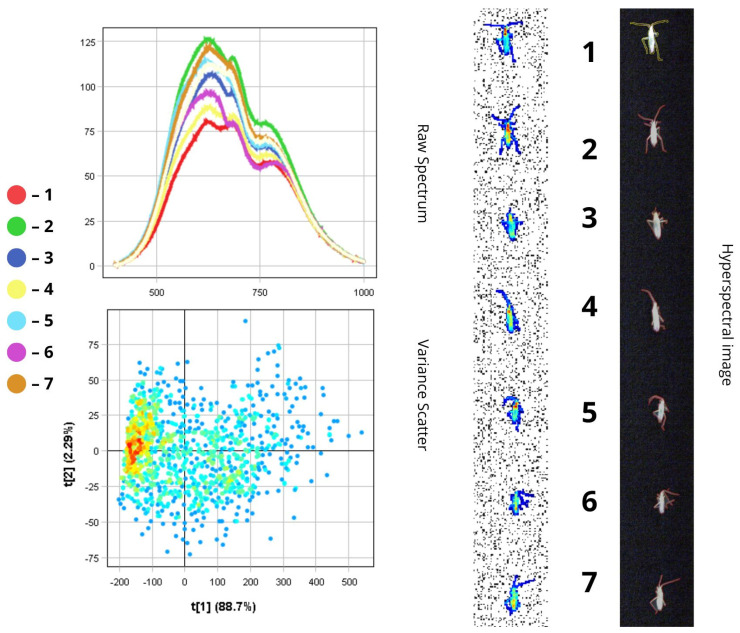
Spectral characteristics of *Trigonotylus ruficornis* extracted from the hyperspectral image.

*Trigonotylus ruficornis* is 6–7 mm long. Its body is pale green or pale yellow, with a matte-textured head and brown eyes (Figure 17). This pale colouration is the main factor contributing to the high reflectance of the integument—exceeding 70% in the visible spectrum (500–700 nm), which is nearly twice as high as that observed in darker-pigmented species.

The raw spectrum graph indicates the highest intensity in the 500–780 nm range, transitioning into the near-infrared region. Peak reflectance values reach 70–75% in the 700–750 nm range, followed by a gradual decrease to about 50–60% in the NIR, suggesting that chitin maintains relatively stable reflectance beyond the visible range. These pronounced spectral peaks result from light reflection off the insect’s pale, chitinous exoskeleton. The matte surface texture, particularly on the head, reduces overall reflectance and is reflected in a more gradual spectral rise and lower peak intensity—by 5–10%—compared to smoother body areas. Minor variations in reflectance may also arise from differences in chitin density or insect orientation, especially in cases where the specimen is ventrally orientated.

The scatterplot shows a tight clustering of pixels, indicating consistent spectral properties across individuals. The first principal component (t[1]) accounts for 88.7% of the variance and is primarily associated with total luminance, while colour tone plays a minimal role in differentiation. The compact distribution of pixel values along the t[1] axis visually confirms this: all spectra are similar in shape, with only slight variations in intensity. Therefore, the high spectral homogeneity observed in *Tr. ruficornis* is a direct result of morphological uniformity—specifically, the absence of structurally distinct features (e.g., wings or contrasting pigmentation zones).

#### 3.1.10. *Chorosoma schillingi*

*Chorosoma schillingi* reaches a length of 11–15 mm and has a slender, yellow-green body. Its distinctive spectral profile results from its light colouration and elongated shape, as reflected in the narrow range of absolute reflectance values across samples—between 33% and 48% in the 550–700 nm peak region—indicating moderate surface optical contrast.

Sample five exhibits the highest reflectance (about 48%) due to its lighter colouration and less dense chitinous exoskeleton, which allows more light to be reflected. In contrast, sample one shows the lowest intensity (about 33%), which is also due to a rougher body surface that increases light scattering. This difference is quantified by an average increase of approximately 10 percentage points in the spectral intensity of sample five relative to sample one across the entire VNIR range. Visually, this is observed as a parallel upward shift in the spectral curve. The spectral plot demonstrates a broad intensity peak within the 500–780 nm range, indicating limited near-infrared reflectance. A key feature of this plot is its flat, wide peak without sharp maxima -typical for matte, light-coloured surfaces with diffuse reflectance.

**Figure 18:**
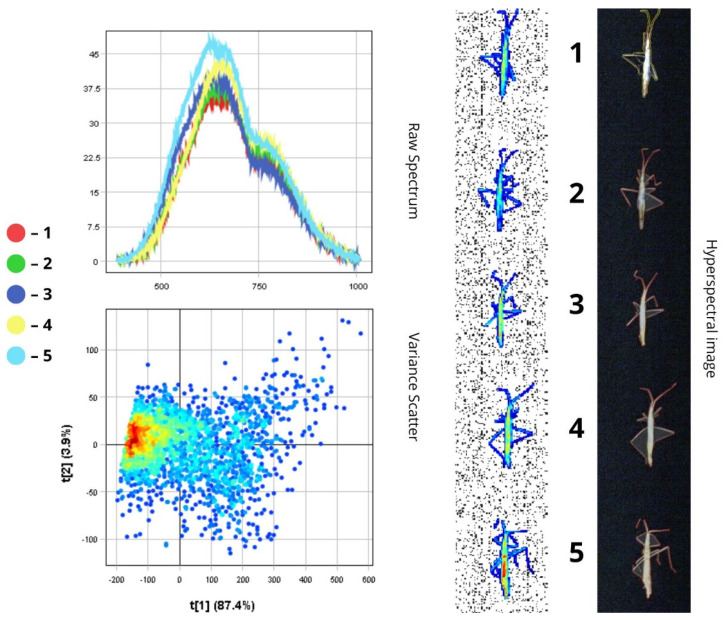
Spectral characteristics of *Chorosoma schillingi* extracted from the hyperspectral image.

In the scatterplot, the moderate point density corresponds to the insect’s narrow body. Narrower anatomical regions offer a reduced surface area, limiting the amount of spectral data that can be captured. However, this does not compromise internal spectral consistency. The principal component t[1], accounting for 87.4% of total variance, shows that even with a relatively small number of pixels, most of the variability is systematic and primarily manifests as differences in overall luminance, which is determined by surface micromorphology (e.g., roughness) and orientation angle. Consequently, the observed moderate data density results from a smaller volume of spectral input and the lower degree of variability inherent to the species’ body structure. Moreover, the elongated body shape may favour localised spectral variations—such as segmentation or minor structural differences—thereby increasing scatter and reducing overall density (Figure 18).

Thus, the compact and elongated pixel cloud distributed along the t[1] axis in the scatterplot indicates that the main source of spectral variability lies in the insect’s surface topology and geometric configuration, as well as luminance differences, rather than pigmentation or colour variation.

#### 3.1.11. *Laodelphax striatella*

**Figure 19:**
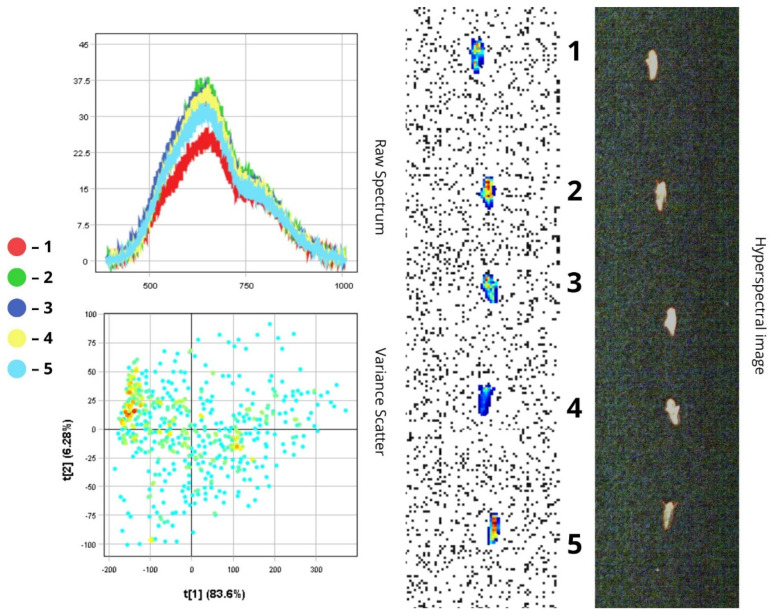
Spectral characteristics of *Laodelphax striatella* extracted from the hyperspectral image.

Adult *Laodelphax striatella* individuals are approximately 4 mm in length. Males have yellow bodies, while females are blackish-brown with distinct black stripes (Figure 19). Female wings are transparent with a brownish tint on the underside, whereas male wings are slightly smoky. Due to this sexual dimorphism, the spectral profiles of males and females may differ depending on pigment composition.

This morphological distinction is clearly reflected in the spectral data: curves corresponding to males (samples 2, 3, and 5) exhibit reflectance values ranging from 33% to 37.5%, while the female spectra (samples 1 and 4) do not exceed 33%, producing a modest contrast of several percentage points. The first sample shows the lowest reflectance (RC ≈ 22.5%), while the second (RC ≈ 37.5%) and third (RC ≈ 37%) have the highest. Specimens orientated ventrally upward tend to exhibit lower intensity (below 25–26%), while those with wings facing upward show higher reflectance (33–37.5%) within the same sex group. The spectral peak is located in the 550–750 nm range. Another noteworthy difference lies in the peak shape: light-coloured individuals display a sharp rise around 550 nm (green region), while darker individuals show a more gradual increase and a shift in the peak toward the red region (~650 nm), which is also due to melanin-induced light absorption.

The low point density in the scatterplot is attributed to the small size of the insect, which limits the quantity of spectral data available. Moreover, the first principal component t[1] accounts for 83.6% of the total variance, clearly indicating that albedo is the dominant structuring factor. The second component, t[2], accounts for only 6.28%, suggesting minimal hue variation within each sex-based colour group. Despite the low data density, the spectral signal is not random noise—it is clearly stratified by sex, further confirming the diagnostic value of hyperspectral data for this species.

### 3.2. Differentiation of Agrocenosis Objects by Hyperspectral Characteristics

**Figure 20:**
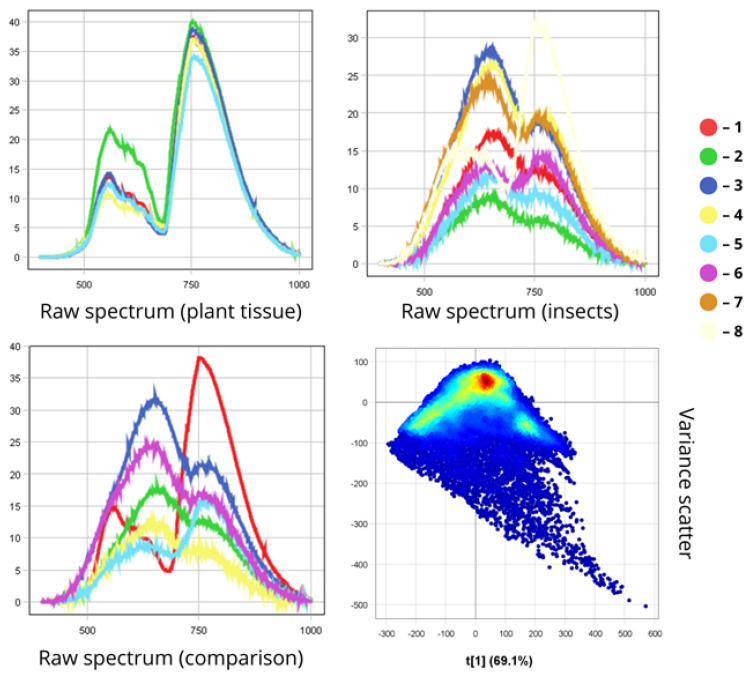
Spectral characteristics of selected pest species compared to plant tissue (1—*Anisoplia austriaca*, 2—*Anisoplia agricola*, 3, 4—*Calliptamus italicus*, 5—*Coccinella septempunctata*, 6—*Tettigonia viridissima*, 7—*Loxostege sticticalis*, 8—*Chrysoperla carnea*).

To demonstrate the spectral differences between insect pests and plant tissue, we analysed the hyperspectral signatures of harmful entomofauna in their natural environment on crop surfaces (Figure 20).

The first graph presents the spectra of plant tissue collected from various foliar organs of spring wheat. All spectral curves show a similar shape, with maximum reflectance occurring in the 750–760 nm range and reaching approximately 40%. At 550 nm (green region), reflectance values lie between 15 and 20%, followed by a sharp increase of 20–25 percentage points from 680 to 760 nm. This corresponds to the well-known “red edge” phenomenon, which is linked to chlorophyll content and the internal leaf structure.

Among the insects, the *A. agricola* leaf beetle shows the lowest reflectance due to its shaded position. In contrast, the lacewing displays the highest reflectance, which is also due to the high reflectance of its transparent wings. The common grain bug also exhibits high reflectance (over 30%), which is attributable to its pale body colouration. Peak reflectance of the lacewing’s integument lies between 30% and 35% in the 750–800 nm range. For darker beetles, these values do not exceed 20%.

The third graph highlights differences in spectral curve shapes between plant tissue and insects. Plant spectra are characterised by a moderate peak near 550 nm and a steep rise at 750 nm (reaching ~40% reflectance). Insect spectra, by contrast, show a pronounced peak around 600 nm and a more subdued increase near 750 nm. Insects exhibit peak amplitudes between 600 and 700 nm, ranging from 10% in darker species to 25–30% in lighter ones. This differs from plants, where reflectance in the near-infrared rises more sharply by 15–20 percentage points. Such variation is one of the primary spectral distinctions between insect integuments and plant tissue. In general, insect reflectance is lower than that of plant tissue, and their spectral curves are more variable. Plant spectra tend to rise sharply and form a stable plateau at longer wavelengths, largely due to chlorophyll content. These curves, especially from healthy leaves, exhibit a smooth and symmetrical shape, reflecting biochemical uniformity. Insect spectra, however, are uneven and contain localised peaks and dips due to chitin structure, wings, and pigment composition. Reflectance is suppressed across the entire spectrum in darker species. Overall, insect spectral curves vary greatly between species, whereas plant curves are more universal.

Clear evidence of this spectral separability is provided by the dispersion scatterplot. The first principal component accounts for 69.1% of total variance, indicating a dominant factor separating insect and plant profiles. High reflectance values in the near-infrared region manifest as a dense, compact point cloud—corresponding to plant tissue due to its uniformity and structural homogeneity. In contrast, more scattered and lower-reflectance points—representing insects—are shifted toward the bottom of the diagram, reflecting their varied capacity to absorb and reflect light. Therefore, the clear separation along the t[1] axis serves as numerical proof of the feasibility of automatic segmentation of these biological objects.

This spectral divergence enables the use of machine learning and hyperspectral data analysis for the automatic identification and classification of insects on crop leaves.

### 3.3. Statistical Analysis of Insect Spectral Characteristics Using Multivariate Methods

A total of 180 morphologically intact and representative insect specimens were selected for hyperspectral imaging (*A. austriaca*—13, *A. agricola*—13, *Ph. fumigata*—16, *Tr. ruficornis*—19, *Ph. vittula*—19, *H. tritici*—20, *Ch. schillingii*—15, *L. sticticalis*—15, *T. viridissima*—10, *Ch. aridula*—15, *C. italicus*—10, *L. striatella*—15), providing a large dataset for training and validating machine learning classification models.

For each insect species (*n* = 10–20), standard errors of the mean (SE) and 95% confidence intervals were calculated. The overall mean standard error across all species was approximately 1.86%, demonstrating an acceptable level of measurement accuracy at a 95% confidence interval. This indicates that the measurements were sufficiently stable and the mean (μ) is reliable. For some species with high variability (e.g., *Ph. fumigata*), the SE may exceed the average, reflecting greater heterogeneity. However, for most species, SE ≤ 2%, indicating moderately high measurement precision.

Statistical analysis of the data enabled the identification of key patterns in insect spectral characteristics, assessment of their variability and stability, and clear presentation of the main results (reflectance/absorption wavelength range and reflection coefficient) (Table 3). The calculated values of the principal statistical parameters are presented in Table 4.

**Figure 21:**
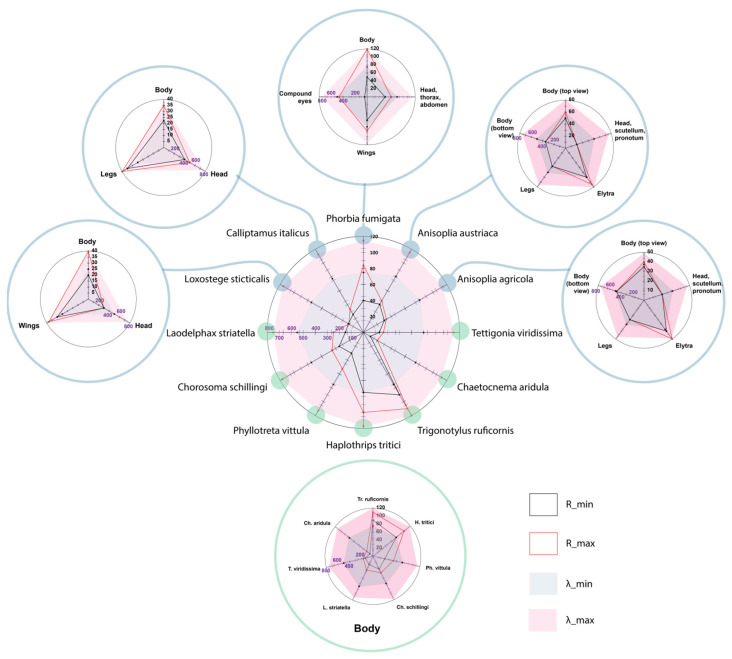
Visualisation of key spectral characteristics of major pest species in the wheat agrocenosis.

The highest maximum reflectance was observed in *Tr. ruficornis* (Rₘₐₓ = 110%) due to the presence of light pigments and in *Ph. fumigata* (Rₘₐₓ = 120%) owing to the reflectance of its wing plate. The lowest reflectance was recorded in *Ch. aridula* (Rₘᵢₙ = 10%) due to its dark pigmentation (Table 4, Figure 21). The average reflectance value (μ) simplifies complex spectral data into a single numeric indicator, facilitating comparisons between individuals or species. *Ch. aridula* has the lowest average reflectance (μ = 15%), which correlates with its low overall spectral intensity. In contrast, *Tr. ruficornis* shows the highest average reflectance (μ = 98.75%), attributed to the presence of light yellow-green pigments. The standard deviation (σ) values across the samples are generally low (average = 7.44%), which confirms the high reproducibility of measurements within a species. For instance, *Ch. aridula* has σ = 2.89% at 750 nm, meaning that most reflectance values fall within ±2.89% of the mean. Higher σ values (up to 36%) may indicate equipment inaccuracies or biological variability, such as within-species variation in pigmentation or morphology. A notable example is *Ph. fumigata*, which shows a maximum deviation of σ = 36%, likely due to high sample heterogeneity. For samples with elevated σ values (e.g., above 20%), additional statistical testing, such as the *t*-test, is recommended for more precise validation. This parameter also offers insight into species variability: a sharp increase in a species’ σ over time may signal adaptation to new environmental conditions.

**Figure 22:**
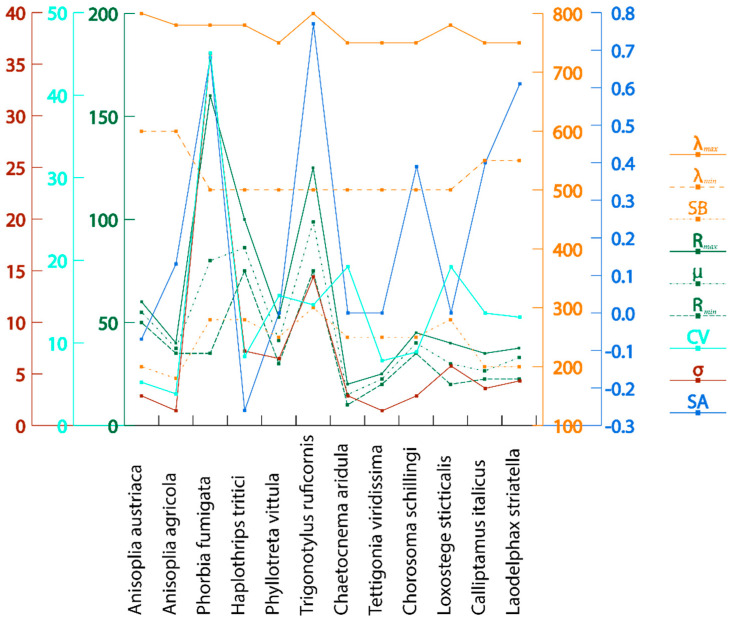
Statistical indicators for wavelength and reflectance values (λₘᵢₙ—minimum wavelength; λₘₐₓ—maximum wavelength; SB—spectral bandwidth; Rₘᵢₙ—minimum reflectance; μ—average reflectance; Rₘₐₓ—maximum reflectance; CV—coefficient of variation; σ—standard deviation; SA—spectral asymmetry).

The visual representation of statistical data is shown in Figure 22. The multivariate statistical analysis reveals complex relationships between spectral parameters and statistical indicators for the studied insect species. To assess the statistical significance of differences in spectral characteristics among the studied pest species, a one-way analysis of variance (ANOVA) was performed based on the averaged data presented in Table 2 and Table 3. The results revealed statistically significant differences between the groups (F = 9.84, *p* < 0.001), confirming the reliability of species-level spectral differentiation. Furthermore, low values of standard deviation (σ) and coefficient of variation (CV) for most samples support the reproducibility of the measurements and the stability of the observed spectral differences.

The observed statistical significance of the differences indicates that the obtained spectral features can be used as reliable diagnostic markers for pest species identification. *Tr. ruficornis* (CV = 14.61%), *Ph. vittula* (CV = 15.74%), *Ch. aridula* (CV = 19.24%), *L. sticticalis* (CV = 19.20%), and *L. striatella* (CV = 13.12%) display moderate variability due to biological heterogeneity—such as age-related differences or cuticle condition. In contrast, *Ph. fumigata* shows a high coefficient of variation (CV = 45.10%)—attributed to both a small sample size and variation in specimen orientation. For instance, one specimen was positioned with its body facing the camera, while another showed its wings, which reflect significantly more light than the darker pigmented body. Species such as *Ph. fumigata* (ΔR = 125%), *H. tritici* (ΔR = 25%), and *L. sticticalis* (ΔR = 20%) exhibit high reflectance deltas due to wing-related interference effects. *Tr. ruficornis* (ΔR = 50%) shows high variability due to differences in age and cuticle condition. In species with darker pigmentation, ΔR typically ranges from 5 to 15%. More resilient species show lower ΔR under changing conditions—for example, *T. viridissima* has a ΔR of 5%, indicating high species stability. Values in the range of 10–20% exceed the natural fluctuations expected in stable populations. For small samples, high ΔR may not be statistically significant, as random variation can distort results. Elevated ΔR and σ values in some species highlight the need to consider external environmental or biological factors in analysis.

Spectral asymmetry (SA) is relatively uniform across all species due to similar chitinous structures, as they all belong to the same class. Slight right skewness is observed in species such as *A. agricola* (SA = 0.13), *Tr. ruficornis* (SA = 0.77), *Ph. fumigata* (SA = 0.68), *Ch. schillingi* (SA = 0.39), *C. italicus* (SA = 0.40), and *L. striatella* (SA = 0.61), likely caused by the presence of rare but intense pigments. Left skewness is observed in *A. austriaca* (SA = −0.07) and *H. tritici* (SA = −0.26), due to melanin content, which absorbs short-wavelength light. Other species display symmetrical spectra. The asymmetry analysis demonstrates that most insects have a red-shifted spectral response, as a phenomenon commonly observed in remote sensing.

### 3.4. Species Identification of Pest Entomofauna from Hyperspectral Data Using a PLS-DA Model

The spectral data served as the foundation for constructing a classification model. Among the available classification methods—PLS-DA and SIMCA—we used the former for species identification of pest entomofauna.

To improve prediction accuracy, we implemented several key optimisation and preprocessing steps as part of our workflow:

(1) Spectral data preprocessing: prior to model training, hyperspectral images underwent spectral data preprocessing. This included extraction of only the regions containing insect specimens to minimise background interference, which effectively reduced noise from irrelevant image areas.

(2) Dimensionality reduction using PCA: a Principal Component Analysis (PCA) model was built using all pixels from the mosaic image. This step served to capture the most informative spectral features while reducing data dimensionality and filtering out noise, which enhances model stability and prediction accuracy.

(3) Model selection and training with PLS-DA: we chose Partial Least Squares Discriminant Analysis (PLS-DA) as the classification method due to its robustness with high-dimensional spectral data and ability to handle multicollinearity. The PLS-DA model was developed based on the refined dataset, focusing on selected insect samples.

(4) Model validation metrics: the model’s predictive quality was evaluated using R^2^ (coefficient of determination), Q^2^ (predictive ability), and RMSEC (root mean square error of calibration), which are indicators of calibration quality and generalisation. These metrics, reported in Table 4, confirm the model’s high predictive power for most species.

(5) Pixel threshold adjustment: in addition, the pixel threshold parameter was adjusted to adapt the model to different insect sizes, allowing better discrimination of both smaller insects (e.g., wheat thrips, flea beetles) and larger ones, thereby improving classification accuracy and reducing the influence of noise.

**Figure 23:**
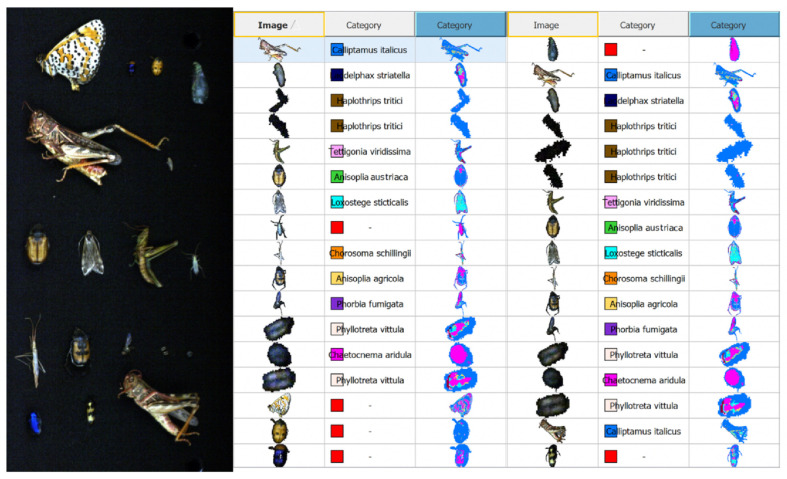
Results of species identification of major wheat agrocenosis pest species using spectral characteristics and the PLS-DA model algorithm.

In summary, noise removal was primarily achieved through targeted spectral preprocessing and pixel region extraction, while optimisation was performed through PCA-based dimensionality reduction, the PLS-DA model settings and pixel threshold adjustments. These combined techniques allowed us to develop a robust and reliable classification model, as reflected in the strong alignment of predicted and observed values shown in Figure 23.

Figure 23 presents the results of species identification based on spectral characteristics as the key classification input.

Visual inspection shows high model performance on the training dataset and a strong alignment between model-detected coloured regions and the actual contours of insect bodies in the image. For species with lower classification accuracy—such as *Ch. aridula* (R^2^ = 0.785)—biological traits play a role in reducing model precision. Specifically, its greenish-bronze, shiny exoskeleton absorbs light due to the dark dorsal pigmentation, resulting in lower reflectance. Combined with its rounded, non-distinctive body shape, this limits the number of unique pixels the model can analyse. As a result, the model may blend *Ch. aridula* with morphologically similar species, reducing spectral distinctiveness.

**Figure 24:**
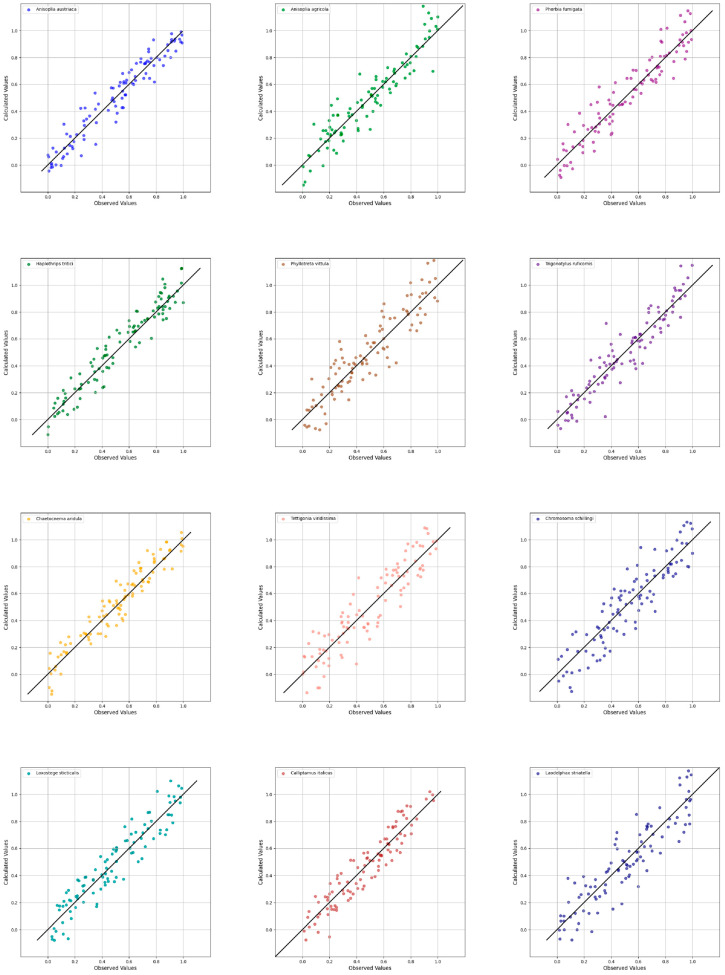
Predicted (*y*-axis) vs. actual (*x*-axis) data distribution using the classification model.

Figure 24 shows the predicted vs. actual distribution of data produced by the classification model. The tight clustering of points confirms the model’s high predictive ability. Point clouds for species like *A. austriaca* (R^2^ = 0.912, Q^2^ = 0.898), *A. agricola* (R^2^ = 0.901, Q^2^ = 0.895), and *T. viridissima* (R^2^ = 0.899, Q^2^ = 0.887) form compact clusters along the ideal prediction line, indicating minimal classification error. Meanwhile, species like *Ch. aridula* (R^2^ = 0.785) and *H. tritici* (R^2^ = 0.799) display more dispersed results, implying a slightly higher prediction error, but still within an acceptable range and demonstrating moderate predictive performance.

By adjusting the pixel threshold, the model can be tailored to identify smaller insects (e.g., thrips, flea beetles) or focus only on larger specimens (e.g., by increasing the threshold to 3000 pixels). Model quality is evaluated using three key metrics: R^2^—coefficient of determination (fit to training data), Q^2^—predictive power of the model, RMSEC—root mean square error of calibration (model error on training set). These values are presented in Table 5.

According to Table 5, the model achieved high classification quality and accuracy for 8 of the 12 species analysed, as indicated by R^2^ > 0.85 and Q^2^ > 0.83. The best results were observed for *A. austriaca* (R^2^ = 0.912, Q^2^ = 0.898, RMSEC = 0.041) and *A. agricola* (R^2^ = 0.901, Q^2^ = 0.895, RMSEC = 0.038). Conversely, the least accurate results were obtained for *Ch. aridula* (R^2^ = 0.785, Q^2^ = 0.761, RMSEC = 0.082) and *H. tritici* (R^2^ = 0.799, Q^2^ = 0.778, RMSEC = 0.076).

Overall, the model demonstrates high classification accuracy for approximately two-thirds of the insect species studied and moderate accuracy for the remaining species. These results are still sufficient for reliable species identification. A distinctive feature of the model is that it does not classify previously unlabelled data or any data not included in the training set. Instead, such inputs are labelled as “Unknown”. This ensures that only the defined and validated species are recognised, confirming the model’s ability to generalise solely based on the spectral characteristics of the samples included in the study. These findings highlight the potential of hyperspectral data as a key diagnostic and monitoring tool for pest species in agroecosystems.

## 4. Discussion

### 4.1. Interpretative Analysis of the Identified Spectral Characteristics of Insects

Based on the obtained spectral data, we identified and described the main patterns and determined the key factors influencing the spectral curves of different insect species, including phenotypically similar ones. This provides a foundation for understanding how hyperspectral imaging can be used to identify insects. To support statements concerning insect morphological structures, bibliographic references are provided; however, all explanations of the observed effects were developed independently, based on the results of our study.

#### 4.1.1. Dependence of Spectral Signatures on the Pigment Composition of Insects

**Figure 25:**
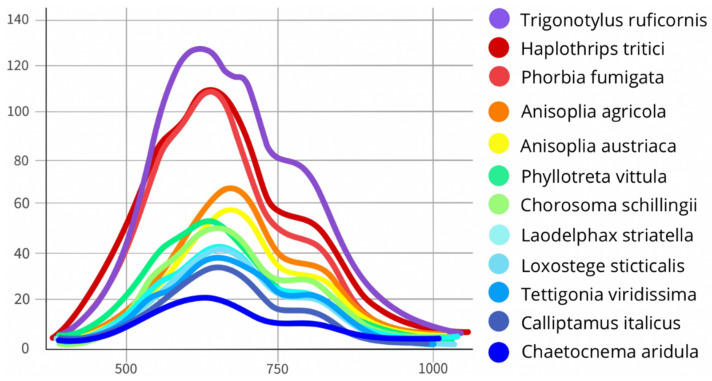
Generalised descriptive spectral features of insects by species.

**Figure 26:**
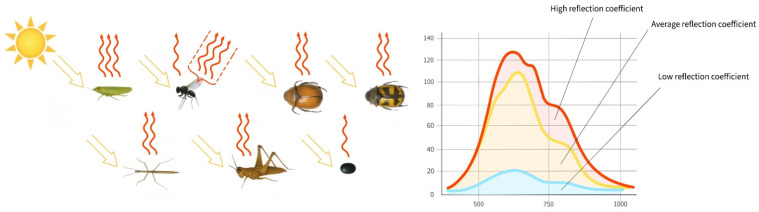
Schematic visualisation of spectral reflectance profiles for various insect species.

Our results are consistent with a study showing that insects with white, red, orange, and yellow colouration exhibit high reflectance, and the same property is also observed in some green species [44]. Lighter areas of the insect body, such as the elytra of the genus *Anisoplia*, the transparent wings of flies and thrips, and the body of grain bugs, show higher levels of reflectance. Bright pigments tend to reflect strongly in the near-infrared range, while smooth surfaces enhance this effect by increasing the reflection coefficient. Conversely, dark body areas rich in melanin absorb more light and therefore have significantly lower reflectance. These areas typically include the legs, head, scutellum, pronotum, thorax, and abdomen. A decrease in spectral intensity can also be attributed to rougher surface textures, the presence of body hairs, and the specific orientation of the insect relative to the light source during imaging. Insects with high melanin content produce the strongest reflectance peaks in the visible range (500–750 nm), while near-infrared reflection is either absent or minimal. The highest reflectance and NIR reflectance are observed in *Ph. fumigata* (due to the reflectance of the wings), *Tr. ruficornis* (due to the light-coloured body), *H. tritici* (due to wing reflection and yellow stripes), and *A. austriaca* and *A. agricola* (due to their large body surface area and the smooth elytra). *A. austriaca* exhibits even higher reflectance due to the absence of dark pigments on the elytra. By contrast, *Ch. aridula* demonstrates the lowest reflectance, primarily due to the absorption of light by dark pigments (Figure 25 and Figure 26). Notably, some insects with pale-coloured surfaces still show relatively low reflectance due to the matte texture of their cuticle. Small body size can also contribute to reduced reflectance intensity.

#### 4.1.2. Influence of Exoskeleton Structure on the Spectral Characteristics of Insects

The formation of species-specific spectral curves is largely influenced by the relief and microstructure of the insect’s exoskeleton, which is especially relevant in the visible and red-edge near-infrared regions.

The structure and texture of the exoskeleton are determined by its molecular composition. Chitin, a key structural component, is made up of β-(1→4)-linked N-acetylglucosamine units, forming strong hydrogen bonds and containing amide, hydroxyl, methyl, and C-H groups. These groups exhibit specific vibrational modes that absorb light in the visible and near-infrared ranges, producing distinct spectral peaks that differentiate chitin from other biopolymers. α-chitin has a compact crystalline structure with antiparallel chains, fewer voids, and low porosity, which likely results in lower reflectance and a broader, less intense spectral peak. β-chitin has a more open structure with parallel chains, allowing interstitial water or air pockets. This promotes internal light reflection and scattering, increasing overall reflectance and producing a more pronounced spectral peak. γ-chitin, with a mixed structure, exhibits intermediate optical properties. Interference effects may occur in localised regions but are less pronounced than in fully parallel arrangements.

**Figure 27:**
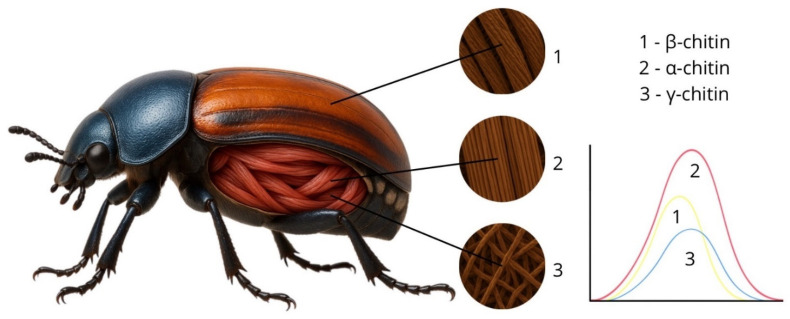
Structural and optical characteristics of crystalline chitin polymorphs and their spectroscopic behaviour.

Internal structures may also influence spectral properties. For instance, peritrophic membranes in some insects may contain γ-chitin, while intestines may include β-chitin. Notably, structural differences in the apodemes are pronounced [58]. The species studied here primarily contain α-chitin, typical of *Coleoptera* and *Orthoptera* families [59], but species with high reflectance likely possess β- or γ-chitin in internal organs such as the digestive system or muscular tissues (Figure 27).

Based on spectral reflectance intensity, it can be assumed that insects with higher reflectance have a higher chitin content. In *Orthoptera*, for example, chitin constitutes up to 8.9% of dry body weight and is of low molecular weight [60], which can affect spectral properties. Furthermore, younger insects may display higher and broader spectral peaks, suggesting that spectral features could be used to estimate insect age. The glossy appearance of the cuticle may result from cuticulin and a waxy layer, as observed in *H. tritici*, which may explain its high reflectance despite high melanin content.

#### 4.1.3. Spectral Reflectance of Insect Body Parts

Compound eyes exhibit moderate reflectance, while the head and abdomen generally reflect the least light due to strong pigment absorption. The reflectance of insect eyes can vary across species because of their multilayered corneal lenses and differing pigment compositions. Most insect eyes contain black screening pigments that block scattered light, reducing background noise in photoreceptor signals [61].

The highest reflectance in *Ph. fumigata*, *H. tritici*, *Tr. ruficornis* and *L. sticticalis*, high reflectance originates from the wing surface. Wings reflect more light than other body parts due to their thin, membranous structure. In *Ph. fumigata*, *H. tritici* and *Tr. ruficornis* the base of the wing is a thin transparent membrane, which naturally exhibits high reflectance intensity. It is noteworthy that the measured reflectance values exceeded 100%, which can be explained by the angle of the objects relative to the light source and the transparent, thin structure of the wings. This creates a specular reflection effect, causing the sensor to register an intensity much higher than the standard level.

**Figure 28:**
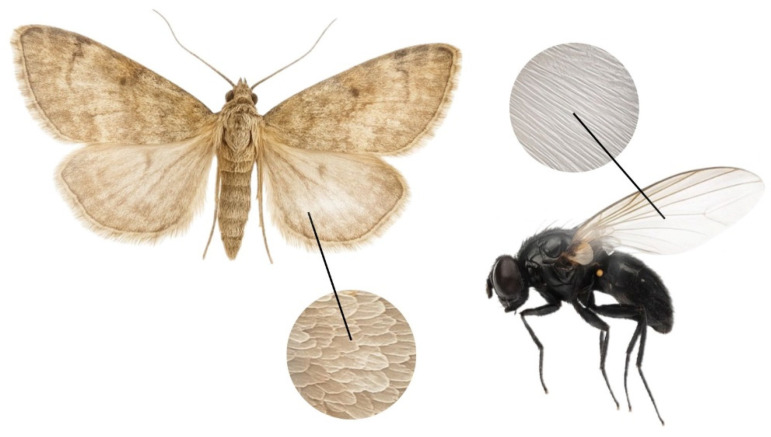
Microstructure of the wings of *Loxostege sticticalis* and *Phorbia fumigata* with high albedo.

In *L. sticticalis*, the wings are covered with venation and tiny scales characteristic of *Lepidoptera*. These scales influence colouration and produce optical effects such as shimmer or iridescence due to interference. Some of these microscopic structures refract and reflect light, significantly increasing wing reflectance—even in the absence of bright pigments. The wing surface, covered in scales, can be microscopically smooth, enhancing reflectance. In many *Lepidoptera*, these scales feature regularly arranged longitudinal ridges formed by stacked lamellae (Figure 28). This morphology leads to pronounced interference and diffraction effects [62]. High wing reflectance may serve adaptive functions, such as preventing overheating by reflecting sunlight, camouflaging via environmental light blending (e.g., sun glare on grass), or increasing visibility for mate attraction.

**Figure 29:**
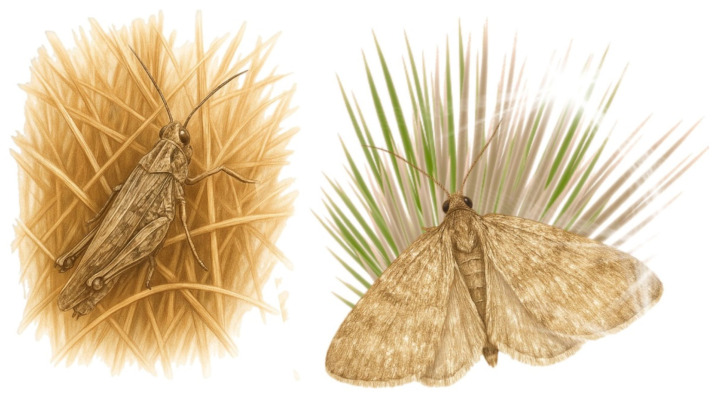
Visual-ecological relationship between the reflectance of insect integuments (*Loxostege sticticalis* and *Calliptamus italicus*) and their adaptation for camouflage in natural landscapes.

In *C. italicus*, the dark colouration of the head may serve as UV protection and camouflage. In contrast, its pale-coloured legs have fewer pigments and reflect more light, helping to prevent overheating during activity. The presence of K_2_O and CaO compounds in *Orthoptera* [63] may further enhance surface reflectance. In summary, as shown in *L. sticticalis* and *C. italicus*, body colouration plays multiple adaptive roles—not only for camouflage against vegetation but also for thermoregulation or UV protection—resulting in distinct colouration across body parts (Figure 29). Thus, the optical traits of living organisms can be interpreted through the lens of evolutionary adaptation.

We found that the spectral characteristics of living organisms are determined by multiple factors and are not limited to a single criterion. Insect optical properties depend on chitin composition and concentration, surface smoothness, pigment types (e.g., carotenoids, melanin), viewing angle, cuticle texture, and even age. Understanding these spectral traits is essential for interpreting the complex and variable optical behaviour of insects—both across and within species, and even within different parts of the same organism.

### 4.2. Interpretive Analysis of PLS-DA Model Performance in the Context of Insect Spectral Variability and Morphometrics

The application of a PLS-DA machine learning model to hyperspectral data for the identification of pest insects in spring wheat agrocenoses demonstrated strong results. However, it is important to emphasise that model performance correlates directly with the distinctiveness of spectral features—shaped by both optical properties of body tissues and species-specific morphological traits.

The highest classification accuracy was achieved for *A. austriaca* (R^2^ = 0.912) and *A. agricola* (R^2^ = 0.901), which have stable and distinct spectral signatures. This is largely due to their matte, dark-coloured cuticle, which provides highly specific reflectance profiles. Species with moderate accuracy, such as *Ph. vittula* (R^2^ = 0.841) and *Tr. ruficornis* (R^2^ = 0.854), show greater spectral variability. This may be linked to morphological heterogeneity—e.g., contrasting colouration in *P. vittula* or the pale, semi-matte cuticle of *Tr. ruficornis*, which is sensitive to changes in lighting and surface relief, creating moderate diffuse reflection. The lowest performance was observed for *Ch. aridula* (R^2^ = 0.785), likely due to the low-contrast integument. Its dark brown, matte surface has weak reflectance, further compounded by strong melanin-based absorption, which reduces spectral variability across body parts.

Body size also significantly affects classification quality. The model successfully identified both large insects (*T. viridissima*, R^2^ = 0.899) and small ones (*H. tritici*, R^2^ = 0.799), indicating that, beyond reflectance, geometric factors such as size and body shape are critically important. Insects with brighter body colours, variable surface relief, larger sizes, and atypical morphology are more easily distinguished by machine learning algorithms, which rely on distinctive “fingerprints” to form unique spectral profiles [64]. Size thresholds for segmenting insects from background also affect classification. When smaller objects must be classified, threshold values should be lowered accordingly. In cases where geometric-morphological features are similar, spectral characteristics become the dominant classification factor.

Despite some discrepancies in the extraction of spectral and geometric features, the model achieved a satisfactory predictive performance, with Q^2^ > 0.76. This confirms the model’s potential for use in monitoring pest populations in agroecosystems. A low RMSEC (0.038–0.041) for species with high R^2^ and Q^2^ values confirms model reliability for these taxonomic groups. While insect detection models have previously been developed (e.g., for aphids [33] or integrated pest identification networks [65,66,67]), this study further demonstrates the robustness of hyperspectral imaging for species-level differentiation in pest monitoring. Future improvements should focus on enhancing classification accuracy for species with weak spectral contrast. This can be achieved by incorporating additional spectral indices and expanding the training dataset to capture broader within-species variability in spectral profiles.

### 4.3. Practical Significance of the Study

The conducted study has significant practical implications for improving the efficiency of monitoring and managing insect pests in agroecosystems. The application of hyperspectral imaging enabled, for the first time, a comprehensive characterisation of the spectral profiles of various spring wheat pest species, revealing patterns of their reflectance depending on pigment composition, exoskeleton structure, and morphological features. Establishing the relationship between morpho-structural characteristics and optical properties of insects, and analysing the influence of chitin type, pigment composition, and surface microtexture on body reflectance, demonstrated species-specific spectral signatures under hyperspectral imaging conditions. This provides a foundation for the development of automated systems for early detection and classification of pests based on optical traits, eliminating the need for manual collection and identification of specimens. The developed PLS-DA model demonstrated high efficiency in identifying individual taxa, opening new prospects for the use of hyperspectral imaging in pest recognition for agricultural crops. Although the study was conducted under laboratory conditions, its results create scientifically grounded prerequisites for the implementation of hyperspectral technologies in agricultural monitoring systems. Implementation requires substantial investment, the use of hyperspectral analysis can significantly increase the accuracy of pest identification and enable timely detection of infestation hotspots, ultimately providing considerable long-term economic benefits.

### 4.4. Economic Efficiency

To assess the practical applicability of the proposed hyperspectral imaging approach, an analysis of its economic efficiency was conducted, considering equipment costs, labour expenses, and potential economic benefits associated with reduced pesticide use and yield loss prevention (Table 6).

The results presented in Table 6 demonstrate the potential economic benefits of implementing hyperspectral pest monitoring. While reductions in yield losses and pesticide use contribute to some economic gain, the high costs associated with equipment, personnel, and preparation of the monitoring environment make the approach economically viable primarily for large-scale operations. Break-even is achievable only when applied over extensive cultivated areas, indicating that widespread adoption would require careful consideration of scale and cost-efficiency. It should be noted that these calculations consider only the basic purchase of a few units of equipment for practical implementation. Simultaneous application of this technology across multiple fields may require additional costs, which would be partly proportional to the area that needs to be surveyed. Nevertheless, even with a basic set of equipment and software, significant positive results can be achieved from the practical implementation of this technology.

## 5. Conclusions

This study presents the first phenotypic characterisation aimed at quantitatively describing and classifying the spectral properties of 12 species of insect pests in wheat agrocenoses using hyperspectral imaging (in the 400–1000 nm range) combined with computer vision technologies. Hyperspectral imaging of the specimens provided detailed spectral data describing various insect pest species. The results demonstrated the high sensitivity of this method to differences in exoskeleton reflectance across species.

Analysis of reflectance spectra revealed that different pest species exhibit distinct spectral properties across specific wavelength ranges, resulting in statistically significant interspecific differences. This variation in spectral intensity reflects differences among body parts and is influenced by morphological features including pigmentation, chitin structure, the presence of a waxy cuticle layer, wing morphology, insect age, body shape, and surface microrelief. Lighter (white, yellow, and green) and smoother areas of the insect body exhibit higher reflectance, while darker regions rich in melanin have lower reflectance. The structure and biochemical composition of the exoskeleton contribute significantly to the formation of species-specific spectral profiles, enabling the creation of reliable spectral profiles for insect identification. Interpretation of these spectral profiles allowed for the identification of distinct spectral signatures characteristic of different groups of phytophagous insects. The highest reflectance was recorded in *Trigonotylus ruficornis*, due to its light yellow-green colouration, and in the wings of *Phorbia fumigata* and *Haplothrips tritici*, which are highly reflective. In contrast, the lowest reflectance was observed in *Chaetocnema aridula*, which is primarily due to light absorption by dark pigments.

Spectral reflectance analysis enables reliable object classification and differentiation. The PLS-DA model developed in this study demonstrated high efficiency in species identification and confirmed the potential of hyperspectral imaging technology for this purpose. The proposed approach effectively discriminated between insect species, underscoring its practical value for agricultural entomology and pest management. For broader implementation of hyperspectral imaging in agriculture, addressing data standardisation and availability. These steps are essential to improve the training of machine vision algorithms for more accurate and consistent insect species identification.

The findings of this study make a significant contribution to advancing the application of hyperspectral imaging for insect detection. The results not only expand the potential use of hyperspectral technologies for pest monitoring in agricultural crops but also support a reduction in insecticide use through more targeted and informed application strategies. As a non-invasive technique, hyperspectral imaging allows for non-destructive analysis of biological specimens and detailed investigation of their structures based on optical properties. Future research will focus on extending the laboratory findings to real field conditions. Planned in situ experiments with hyperspectral imaging of living insects on wheat plants under natural light will help evaluate the model’s robustness and applicability in practical agroecosystem monitoring. Looking ahead, this technology could be integrated with drones and high-resolution cameras, enabling large-scale monitoring of agricultural fields, early detection of infestations, and prediction of pest spread patterns. Consequently, the integration of hyperspectral imaging with UAV platforms could become a cornerstone of precision agriculture.

This study has economic relevance within the broader context of digital transformation in agriculture and the increasing adoption of precision farming systems. Hyperspectral imaging has the potential to improve food security and crop productivity, optimise farm management practices, reduce operational costs, and minimise the environmental impact of farming by enabling targeted insecticide application.

We acknowledge that the practical and economic challenges of implementing hyperspectral pest monitoring—such as equipment costs, preparation of the monitoring environment, and required technical expertise—limit its immediate large-scale adoption. This study demonstrates the effectiveness of the concept, providing a basis for future research to focus on optimising costs and workflows, as well as evaluating scalability for broader practical implementation.

In conclusion, this research provides a foundation for the development of automated insect monitoring systems with species identification accuracy reaching up to 85–90%, which aligns with the ongoing digitalisation of the agricultural sector. Therefore, hyperspectral methods may play a pivotal role in the transition toward more sustainable, data-driven agriculture. Future studies will aim to enhance the classification of species exhibiting lower spectral contrast or smaller size by employing advanced feature extraction techniques and alternative machine learning algorithms.

## Figures and Tables

**Table 1:** Classification and rationale for selecting the insect species as study objects.

No.	Research Object	Insect Category	Impact on Grain Crops	Justification for Selection
1	*Anisoplia austriaca*	Pest of grain crops	Feeds on grains, damages ears, reduces crop yield	Widespread in cereal fields and of significant economic importance
2	*Anisoplia agricola*	Pest of grain crops	Feeds on grains during the grain-filling stage	Economically significant, especially during population outbreaks in dry periods
3	*Phorbia fumigata*	Pest of grain crops	Larvae damage the underground part of the stem, causing wilting of young seedlings	A dangerous pest during the early seedling stage
4	*Trigonotylus ruficornis*	Pest of grain crops	Sucks sap from stems and leaves, disrupting photosynthesis and plant growth	Significantly reduces grain crop yield
5	*Phyllotreta vittula*	Pest of grain crops	Chews on leaves, leading to seedling damage	A serious threat during the early growth stages of crops
6	*Haplothrips tritici*	Pest of grain crops	Feeds on grains within ears, reducing their quality and weight	Mass outbreaks cause substantial yield losses
7	*Chorosoma schillingii*	Pest of grain crops	Degrades grain quality by extracting sap from ears and stems	Causes “empty-ear” syndrome
8	*Loxostege sticticalis*	Polyphagous pest	Causes leaf skeletonisation	Outbreaks can result in complete destruction of crops
9	*Tettigonia viridissima*	Polyphagous pest	Damages leaves and stems	Reduces photosynthetic activity by damaging vegetative biomass; high local densities reduce yield
10	*Chaetocnema aridula*	Pest of grain crops	Larvae damage roots and stems; adults feed on leaves	Causes both above- and below-ground damage, especially severe during drought conditions
11	*Calliptamus italicus*	Polyphagous pest	Consumes aerial parts of plants, including crops	Outbreaks can devastate large crop areas and disrupt structures of agricultural landscapes
12	*Laodelphax striatella*	Pest of grain crops	Damages phloem and transmits viruses	Weakens plants through direct feeding and virus transmission, disrupting metabolism and reducing yields

**Table 2:** PCA results for pest insect spectral data (6 principal components).

Principal Component	Eigenvalue	Explained Variance (%)	Cumulative Variance (%)
PC1	6.82	66.0	66.0
PC2	1.50	14.4	80.4
PC3	0.80	7.7	88.1
PC4	0.55	5.3	93.4
PC5	0.40	3.8	97.2
PC6	0.35	2.8	100.0

**Table 3:** Spectral characteristic parameters of insects’ body parts.

No.	Species	Body Part	Wavelength (nm)	Reflection Coefficient (%)
1	*Phorbia fumigata*	Body	500–780	50–120
		Head, thorax, abdomen	500–750	45–60
		Wings	500–780	58–85
		Compound eyes	500–750	60–70
2	*Trigonotylus ruficornis*	Body	500–800	90–110
3	*Haplothrips tritici*	Body	500–780	75–100
4	*Anisoplia austriaca*	Body (top view)	600–800	50–60
		Head, scutellum, pronotum	500–750	20
		Elytra	600–800	60–75
		Legs	500–750	37.5
		Body (bottom view)	500–750	35
5	*Anisoplia agricola*	Body (top view)	600–780	35–40
		Head, scutellum, pronotum	500–750	20
		Elytra	600–780	40–50
		Legs	500–750	25
		Body (bottom view)	500–750	30
6	*Phyllotreta vittula*	Body	500–750	30–52.5
7	*Chorosoma schillingi*	Body	500–780	35–45
8	*Loxostege sticticalis*	Body	500–780	20–40
		Head	500–750	15
		Wings	500–780	30–38
9	*Laodelphax striatella*	Body	550–750	22.5–37.5
10	*Calliptamus italicus*	Body	550–750	22.5–35
		Head	500–750	20–25
		Legs	500–750	35–40
11	*Tettigonia viridissima*	Body	500–750	20–25
12	*Chaetocnema aridula*	Body	500–750	10–20

**Table 4:** Statistical indicators of insects (reflection and wave range).

No.	Pest Species	Reflection Coefficient, %							Wave Range, nm			
		$R_{min}$	$R_{max}$	μ	σ	CV	Mкp	∆R	$\lambda_{min}$	$\lambda_{max}$	SB	SA
1	*Anisoplia austriaca*	50	60	55	2.88	5.24	55	10	600	800	200	−0.07
2	*Anisoplia agricola*	35	40	37.50	1.44	3.84	37.50	5	600	780	180	0.13
3	*Phorbia fumigata*	35	160	80	36	45.10	80	125	500	780	280	0.68
4	*Haplothrips tritici*	75	100	86.25	7.21	8.36	86.25	25	500	780	280	−0.26
5	*Phyllotreta vittula*	30	52.50	41.25	6.49	15.74	41.25	22.50	500	750	250	0
6	*Trigonotylus ruficornis*	75	125	98.75	14.40	14.61	98.75	50	500	800	300	0.77
7	*Chaetocnema aridula*	10	20	15	2.88	19.24	15	10	500	750	250	0
8	*Tettigonia viridissima*	20	25	18.40	1.44	7.84	18.40	5	500	750	250	0
9	*Chorosoma schillingi*	35	45	32.40	2.88	8.90	32.40	10	500	750	250	0.39
10	*Loxostege sticticalis*	20	40	30	5.77	19.20	30	20	500	780	280	0
11	*Calliptamus italicus*	22.50	35	26.50	3.60	13.60	26.50	12.50	550	750	200	0.40
12	*Laodelphax striatella*	22.50	37.50	33	4.33	13.12	33	15	550	750	200	0.61

**Table 5:** Quality indicators of the trained model (Bright green—highest performance; pale green—high performance; blue and yellow—moderate performance. Blue: R^2^ > 0.8; Yellow: Q^2^ < 0.8).

No.	Pest Species	R^2^ (Coefficient of Determination)	Q^2^ (Predictive Power of the Model)	RMSEC (Expected Calibration Error)
1	*Anisoplia austriaca*	0.912	0.898	0.041
2	*Anisoplia agricola*	0.901	0.895	0.038
3	*Phorbia fumigata*	0.875	0.852	0.053
4	*Haplothrips tritici*	0.799	0.778	0.076
5	*Phyllotreta vittula*	0.841	0.815	0.065
6	*Trigonotylus ruficornis*	0.854	0.831	0.061
7	*Chaetocnema aridula*	0.785	0.761	0.082
8	*Tettigonia viridissima*	0.899	0.887	0.039
9	*Chorosoma schillingi*	0.855	0.833	0.069
10	*Loxostege sticticalis*	0.865	0.844	0.056
11	*Calliptamus italicus*	0.888	0.876	0.045
12	*Laodelphax striatella*	0.812	0.795	0.071

**Table 6:** Estimated economic efficiency of hyperspectral pest monitoring implementation.

Parameter	Value	Unit
Average grain yield	15.2	quintal/ha
Average cost per quintal of grain	14.5	USD
Gross revenue per hectare	220.4	USD/ha
Cost of insecticides	100,000	USD/year
Insecticide cost per hectare	7.2	USD/ha
Basic equipment and software cost	100,000	USD
Personnel and data processing	40,000	USD
Preparation and operation of environment	220,000	USD
Additional costs (amortisation, taxes, maintenance, etc.)	50,000	USD
Total implementation cost	410,000	USD
Expected reduction in yield losses	10	%
Expected reduction in insecticide use	10	%
Total economic benefit per hectare	23	USD/ha
Break-even area	17,826	ha
Return on investment (ROI)	1.0	ratio

## Data Availability

The original contributions presented in the study are included in the article. Further inquiries can be directed to the corresponding author.
